# Using objective measures of physical activity, sleep, and breathing for disease profiling of patients with systemic lupus erythematosus and Sjögren's disease

**DOI:** 10.3389/fdgth.2026.1759967

**Published:** 2026-05-29

**Authors:** Mehdi Boukhechba, Zhi Li, Elena Reynoso, Ioannis Pandis, Kenneth Mosca, Mark Morris, Stefan Avey

**Affiliations:** Johnson & Johnson, New Brunswick, NJ, United States

**Keywords:** digital health, disease profiling, sensing, sjögren's disease, systemic lupus erythematosus

## Abstract

**Background:**

Patients with Systemic Lupus Erythematosus (SLE) and Sjögren's disease (SjD) often report disrupted sleep, excessive fatigue, and decreased physical activity. Symptom assessment and their impact on daily life relies heavily on subjective measures, which are limited. Investigating novel digital biomarkers could facilitate continuous monitoring of symptoms such as sleep disturbances and reduced physical capacity to better capture disease impact and therapy effects.

**Objective:**

To evaluate passive sensing for identifying activity, sleep, and breathing patterns that distinguish SLE and SjD phenotypes in the home environment.

**Methods:**

29 SLE, 29 SjD and 37 demographic-matched healthy participants were recruited in a 6-month in-home study**.** We investigated a set of digital measures collected from a wrist worn actigraphy device (ActiGraph CentrePoint Insight Watch) to measure physical activity and a wall mounted non-contact radio wave device (Emerald) to measure sleep staging and breathing signals during sleep. In addition, self-reported eDiaries and clinical assessments of disease activity were administered. We performed a disease profiling analysis by exploring how eDiary and digital data differ by cohort. We also investigated how ActiGraph and Emerald data correlate with eDiary and disease activity.

**Results:**

Results from ActiGraph and Emerald data suggest that SLE participants exhibited lower physical activity, poorer sleep quality, and higher breathing rate and breathing variability when compared to healthy participants. Similarly to SLE participants, SjD participants showed a reduction in physical activity with an earlier peak activity time, but no differences were recorded in sleep and breathing. Overall, many of the digital measurements of physical activity, sleep, and breathing had weak correlation with self-reported symptoms captured via eDiary and both higher breathing rate and breathing variability were associated with higher SLE disease activity.

**Conclusions:**

Data obtained from digital health devices indicates that physical activity is disrupted in patients with SLE and SjD, while sleep and breathing patterns are also impaired in those with SLE. These results align with the known SLE and SJD symptoms that affect physical activity and sleep and provide initial support for the importance of using passive sensing to understand quality of life in individuals living with chronic autoimmune diseases.

## 1 Introduction

The prevalence of autoimmune diseases has garnered significant attention in the medical community due to its substantial impact on global health. A recent population-based study of 22 million people in the UK shows that autoimmune disorders now affect about one in ten individuals (1). These diseases, characterized by the immune system's misdirected attack on the body's own cells, tissues, and organs, have become increasingly common and affect millions of people worldwide.

Systemic rheumatological diseases such as Systemic Lupus Erythematosus (SLE) and Sjögren's Disease (SjD) are among the most common autoimmune diseases affecting millions worldwide. Disrupted sleep and reduced physical activity are some of the top complaints of patients with SLE and SjD (2–4). Current methods for assessing symptoms of SLE and SjD and their impact on daily life are mainly based on subjective measures that are known to be sensitive to factors such as the respondent's mood, cognitive abilities, and personal biases, which can affect the consistency and validity of the reported data (5). Exploring novel methods to fill these gaps and provide a low burden, unobtrusive, and continuous means for assessing symptoms of SLE and SjD and their impact on daily lives is essential.

Emerging research suggests that patient well-being can be assessed in an objective manner by using remote sensing technology. Today, ubiquitous mobile technologies and small, unobtrusive sensors make it possible to capture streaming digital data that reports aspects of a patient's physiology, behavior and symptoms both quantitatively and in real time (6). As a result, it may be possible to develop streaming disease readouts that are more accurate and less obtrusive than relying on patient and caregiver reports alone (6–8)

Multiple studies have used actigraphy to objectively evaluate sleep deficiency in patients with SLE and SjD. Collectively, these works report evidence of impaired sleep quality and altered sleep patterns when compared with controls (9–16). In SLE, actigraphy frequently shows reduced sleep maintenance (lower sleep efficiency) and increased wake after sleep onset (WASO), with some cohorts also showing longer total sleep time, which may reflect compensatory sleep or disease-related hypersomnia (9–11, 14–16). In SjD, findings are more heterogeneous: several studies document substantial subjective sleep disturbance and fatigue, yet objective actigraphy often reveals no or only modest differences in total sleep time, sleep efficiency, or awakenings when comparing SjD patients to controls (12, 13). Across the body of work, methodological limitations temper the strength and generalizability of conclusions. Common constraints include small sample sizes that are typically underpowered to detect modest differences in several sleep endpoints, cross-sectional designs that limit causal inference, and relatively short monitoring windows (most studies collect data for one to two weeks, with some as short as 7 days), which may fail to capture longer-term variability in sleep. In addition, many studies do not comprehensively assess daytime activity or mobility, which constrains interpretation of the broader impact of sleep disturbances on daily functioning. Consequently, there is a clear need for larger, longitudinal, multiregional studies that employ standardized actigraphy protocols, longer monitoring periods (beyond 14 days), and parallel daytime activity measures to more precisely characterize sleep-deficiency phenotypes and their clinical correlates in SLE and SjD.

The aim of this study was to identify distinct patterns of activity, sleep and breathing parameters in the home environment that are indicative of disease burden in patients with SLE or SjD. These digital biomarkers could be useful for continuously monitoring symptoms like sleep and mobility disturbance to better understand the disease impact and the effect of therapy on patients' daily lives. This work is important to inform future development of remote symptom monitoring through ubiquitous sensing that could be used to assess disease trajectory and recovery over time.

In a cohort including healthy, SLE and SjD participants, longitudinal digital health data were collected through an actigraphy and a radio-based touchless sensor to collect markers of physical activity, sleep and breathing. The data obtained from SLE and SjD patients were compared with that of demographic-matched healthy controls to better understand the disease underlying differences. We also evaluated the correlation of these digital measures with self-reported symptoms from the eDiary and with disease severity to explore the usefulness of collecting these measurements as novel noninvasive readouts describing patient well-being.

This work builds on top of existing works in several ways. An increased sample size and an extended data collection period compared to published studies allow more robust evaluation of disease associated changes in sleep and activity metrics. Additionally, to the best of our knowledge and aside from our recent paper published on a subset of data from this same study (17), this is the first work combining non-contact sensing and actigraphy in SLE and SjD to explore sleep, physical activity and physiology simultaneously.

## 2 Methods

### 2.1 Study design

We conducted a direct-to-patient, prospective, longitudinal, non-interventional, exploratory study for digital biomarker data profiling in healthy normal volunteers (HNV) and participants with Systemic Lupus Erythematosus (SLE) and Sjögren's Disease (SjD). The study was approved by an independent institutional review board [Advarrar (18)] and all experiments were performed in accordance with relevant guidelines and regulations. The study was conducted in the United States between October 2019 and January 2022. In this study, we used the Network Oriented Research Assistant (NORA) platform, Science37's proprietary, cloud-based digital platform designed to enable decentralized, direct-to-patient clinical trials. NORA supports remote participation by integrating a participant-facing mobile application with telemedicine capabilities, electronic consent, and digital data capture, allowing many study activities to be conducted outside traditional clinical research sites. Through secure mobile and web interfaces, participants can complete questionnaires and ePROs, submit study data, receive reminders, and communicate with study staff via messaging and video visits, while researchers remotely monitor study progress and data collection through a web-based dashboard. By serving as the technological backbone of Science37's decentralized trial model, NORA is intended to reduce participant burden, expand access to clinical research regardless of geography, and support efficient, patient-centric study conduct.

*N* = 99 participants were recruited in a 6-month, longitudinal, non-interventional study for digital measure data profiling in healthy (*N* = 39), SLE (*N* = 30) and SjD (*N* = 30) participants. Recruitment was performed by Science 37 (19) following their virtual recruitment methods (e.g., social media, e-mail campaigns, online advertising) and all study participants provided informed consent. The sample size was based on a planned 100 participants including 40 HNV, 30 participants with SLE, and 30 participants with SjD. The sample size selection was guided by the desired precision of the confidence intervals for estimates of sleep efficiency and sleep fragmentation. Based on previous studies in SLE patients (9, 10), a sample size of 30 patients and 40 HNV was expected to provide non-overlapping 95% confidence intervals for the estimation of sleep efficiency and sleep fragmentation index when the effect size was as small as a 1 unit difference between groups.There was no therapeutic intervention in this study and the protocol did not restrict or introduce any medical interventions, including medications.

Participants were all identified based on their medical history records through Science 37 (19). For HNV, participants must be healthy by medical history at screening (No sleep apnea, diabetes, hypertension, cardiovascular disease, Crohn's disease). SjD participants must be diagnosed with primary Sjögren's Disease according to ACR/EULAR classification with extraglandular systemic involvement and must provide evidence of current active extraglandular involvement at screening (20). For SLE, participants must have a history of SLE with evidence meeting SLICC modification of the ACR criteria and showing current active systemic involvement at screening with SLEDAI-2 K ≥ 6 (21). Confirmation of diagnosis was performed by trained physicians reviewing documented medical history (including historical lab records), disease indices, and study-specific lab assessments.

**Figure 1:**
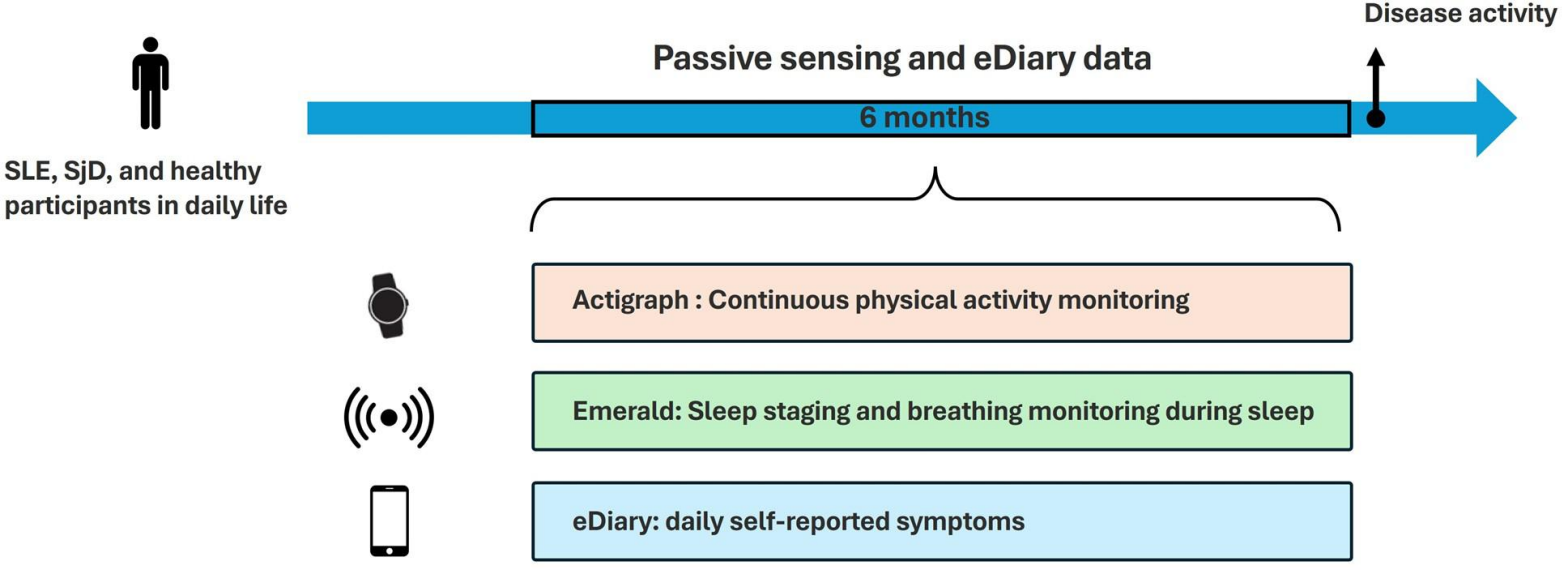
Overall study design.

The total duration of participation in this study was 7 months, including up to a 28- day screening period (see Figure 1). The purpose of the screening period was to confirm eligibility by a healthcare professional (mobile nurse) exam and mobile phlebotomy for laboratory measurements (including those used in the disease scoring). Physical activity and sleep parameters were captured throughout the 6 months of the study using wrist-worn actigraphy devices [ActiGraph CentrePoint Insight Watch (22)] and a non-contact wall-mounted radio wave device [Emerald (23)]. In total, 99 participants were enrolled. The primary study objective was to identify differences in activity metrics using a wrist-worn actigraphy device and Emerald was later added to the study for in-depth sleep staging and breathing monitoring via protocol amendment. Consequently, all participants wore a wrist-worn actigraphy device, and a subset (*n* = 70) had an Emerald sensor deployed in their home bedroom.

To prevent seasonal bias between cohorts known to impact many sleep and activity measures, the study recruited SLE and SjD patients and then identified healthy volunteers to match the patient population on a rolling basis. Healthy participants were demographically matched on biological sex (male/female), age at pre-screening (within +/- 5 years), Body Mass Index (BMI) at pre-screening (within +/- 5 BMI). In cases of multiple pre-screened healthy matches, further matching was done on geographic region (3 regions within United States), race, closest age, closest BMI. Matching based on race was performed to minimize systematic bias from both genetic and non-genetic factors (environmental/lifestyle) that could impact results (24).

While all enrolled patients were demographically matched to a healthy volunteer, only two-thirds of the matched healthy volunteers were enrolled in the study to meet the pre-determined healthy volunteer sample size.

#### 2.1.1 Exclusion criteria

The study was designed to limit the presence of the confounding factors that could influence sleep, mobility, or psychiatric status. As such. any potential participant who met any of the following criteria were excluded from participating in the study:

1. Has an acute or chronic infectious illness (example: human immunodeficiency virus, hepatitis B or C virus, tuberculosis, opportunistic infections) based on medical record at the time of screening.
2. Received an investigational intervention (including investigational vaccines) or used an invasive investigational medical device within 3 months prior to screening or intend to use during the study.
3. History of liver or renal insufficiency (except disease related insufficiency in SLE or SjD participants); significant cardiac, vascular, pulmonary, gastrointestinal, endocrine, neurologic, hematologic, rheumatologic, psychiatric, or metabolic disturbances.
4. Bedridden, immobile or planned to have major surgery within the study timeframe
5. Taking sleeping pills such as, but not limited to, over the counter sleep aids, (Ambien®, Ambien® CR (zolpidem tartrate), Dalmane®, (flurazepam hydrochloride), Halcion® (triazolam), Lunesta® (eszopiclone), Prosom® (estazolam), Restoril® (temazepam), Rozerem® (ramelteon), Silenor® (doxepin) for insomnia or any other sleep disorder.
6. Any condition for which, in the opinion of the investigator, participation would not be in the best interest of the participant (e.g., compromise the wellbeing) or that could prevent, limit, or confound the protocol-specified assessments
7. History of medical illnesses that would prevent, limit or confound the participant’s ability to comply with all aspects of the study.
8. Pets in the bed. The presence of pets can compromise Emerald’s capacity to reliably record participants’ movement and respiratory patterns

### 2.2 Digital health data collection

#### 2.2.1 Actigraph CentrePoint insight watch (CPIW)

The ActiGraph CPIW device (22) is a wrist-worn device that records continuous, high-resolution acceleration. The device was programmed to collect accelerometer data at 32 Hz frequency. While CPIW can measure skin temperature, it was not enabled in this study to preserve battery life and storage capacity. Study participants were asked to wear the CPIW on the non-dominant wrist. Participants were instructed to charge the CPIW every 3 weeks and charging usually takes 2–3 h. Data from the CPIW were transferred from the watch to the Centrepoint Data Hub via Bluetooth and then data were transferred to the CentrePoint cloud by cellular-enabled hubs. Raw data were transferred during physical docking of the watch to the hub (also used for charging).

Participants were asked to wear the actigraphy watch on their non-dominant wrist 24 h a day. Participants were asked to remove the actigraphy watch for any water activity that would exceed 1 meter of depth for more than 30 min as well as for charging every 3 weeks. To ensure adequete data coverage, compliance was monitored to identify participants with low compliance rates. Compliance was measured weekly and participants having less than 75% of days with more than 20 h of wear per day would receive a phone call from Science 37 to remind them about the instructions for wearing the watch.

#### 2.2.2 Emerald

**Figure 2:**
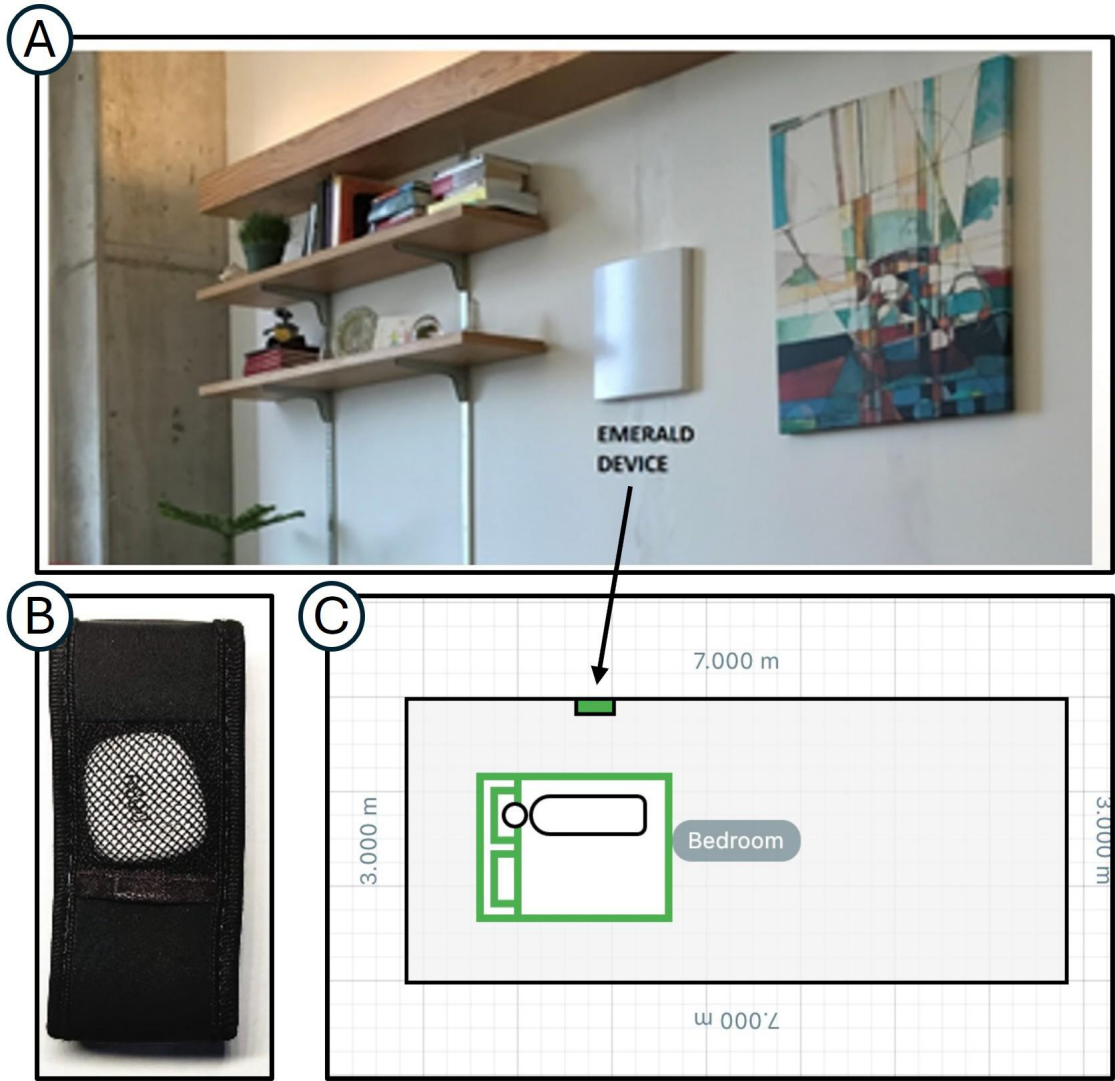
**(A)** emerald RF sensor. The sensor was mounted on a bedroom wall within typically 2-3 meters of the bed. **(B)** An ankle accelerometer was worn for the first 14 days of the study to associate the RF signals with the study participants. **(C)** Example of layout of a participant's bedroom with the Emerald device (green box) installed on the wall.

We used the Emerald device (23) to remotely capture breathing and sleep patterns in patients' natural environment. The Emerald radio frequency (RF) sensor was mounted on a bedroom wall on the side of the bed on which the participant sleeps within 2–3 m of the bed. The sensor did not require the participants to interact with it. In some cases, the wall was not suitable for mounting, and the sensor was mounted on a portable stand instead. Study participants agreed to sleep on a designated side of the bed with no other people allowed on their side of the bed and no pets allowed in the bedroom to ensure the algorithms were able to accurately quantify sleep and breathing (see Figure 2).

The Emerald system consists of a general-purpose wireless sensor that transmits RF signals and then captures their reflections. The Emerald sensor employs frequency-modulated continuous-wave (FMCW) radar and antennae arrays and receives reflections from nearby people (25). The high degree of water content in the human body (∼60%) facilitates the reflection of the radio signals and modulates them with the person's movements (26, 27). The Emerald RF sensor is manufactured by Emerald Innovations, Inc (23). and the dimensions of the sensor are 30 × 35 × 5 cm (Figure 2).

**Figure 3:**
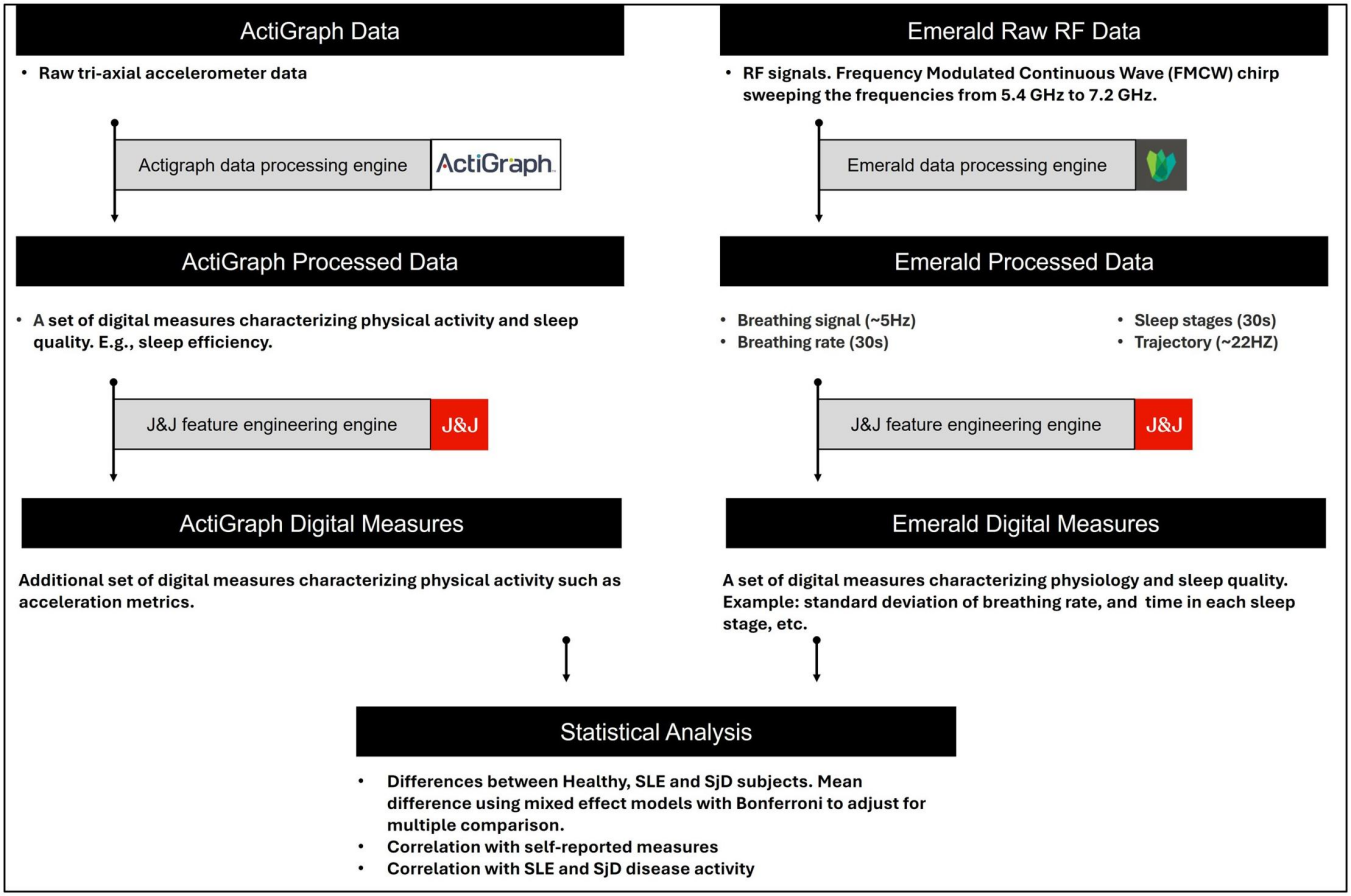
Our pipeline to process actigraphy and emerald data.

As depicted in Figure 3, the Emerald system processes the raw RF signals, which is constituted of a Frequency Modulated Continuous Wave (FMCW) chirp sweeping the frequencies from 5.4 GHz to 7.2 GHz. Details of how Emerald is extracting these signals and the accuracy of their measurements can be found in previous research (26–28). Emerald's system extracted breathing signal at 5 Hz (body displacement due to breathing), breathing rate every 30 s, sleep stages every 30 s [i.e., awake, light, Rapid Eye Movement (REM), and deep sleep], and trajectory data at 22 Hz which represent time series of (x, y) coordinates of the subject when they are moving within the range of the Emerald Device (the trajectory data are not discussed in this paper). Note that because the Emerald sensor placement was chosen to focus on sleep (2–3 m from the bed), measures of breathing are only captured during in-bed periods. The collected Emerald RF data are compressed and transmitted after encryption via Wi-Fi to a secure server.

### 2.3 Digital health data processing

Figure 3 presents our methods for processing the data collected from the two digital health technologies deployed in this study. Streams derived from the two devices are processed to extract digital measures (Table 1) and to analyze differences between HNV, SLE, and SjD participants.

**Table 1:** The self-reported symptoms, disease activity, and a representative set of digital measures of physical activity, sleep, and breathing extracted in this study. .

Type	Source	Measure	Frequency	Description
Clinical assessment and self- reported measures				
Self-reported symptoms	Self-reported eDiary	Fatigue	Daily	Max fatigue in the last 24 h. 0 to 100 with higher values indicating more fatigue.
		Pain	Daily	Max pain in the past 24 h. 0 to 100 with higher values indicating more pain.
		Unable to perform everyday tasks	Daily	0 to 100 with higher values indicating more inability to perform everyday tasks.
		Thinking clearly	Daily	0 to 100 with higher values indicating more clarity in thinking.
		Satisfied with sleep	Daily	0 to 100 with higher values indicating more satisfaction with sleep.
		Feeling depressed	Daily	0 to 100 with higher values indicating feeling more depressed.
Disease Activity	Clinical assessment by trained personnel	SLEDAI	At the end of the study	Systemic Lupus Erythematosus Disease Activity Index 2000. 0 to 105 with higher values indicating more severe disease activity.
		ESSDAI	At the end of the study	EULAR Sjögren's Disease Activity Index. 0 to 36 with higher values indicating more severe disease activity.
Digital measures				
Physical Activity	Actigraph device			
		Average acceleration	Daily	The average acceleration value measured by ENMO during the day.
		Start time of the 10 most active hours	Daily	The number of hours since midnight of the start time of the most active 10 h of the day.
		Start time of the 5 least active hours	Daily	The number of hours since midnight of the start time of the least active 5 h of the day
		Intra-daily variability	Daily	The relative fragmentation within days in the rest-activity pattern. Higher values indicate greater fragmentation.
		Relative amplitude	Daily	The relative difference in activity level between the 10 most active and five least active hours.
Sleep	Emerald device	Duration of sleep stages	Daily	Time spent awake, in light, deep, or REM sleep
		Sleep efficiency	Daily	The amount of time the participants spend asleep while in bed
		Entropy of sleep stages	Daily	Sample entropy of sleep stages
		Average sleep segments	Daily	The average duration of sleep segments in hours.
		Total duration of sleep interruptions	Daily	The total duration of sleep interruptions in minutes.
		Number of Sleep Interruptions	Daily	The number of sleep interruptions per night.
		Sleep Midpoint Time	Daily	The midpoint of sleep (MSF) is the clock time halfway between sleep onset and waking up measured in hours.
		Total Sleep Opportunity	Daily	The total sleep opportunity is the time between sleep onset and wake up in addition to sleep onset latency measured in hours.
Breathing	Emerald device	AVG breathing rate	Daily	Average breathing rate
		AVG breathing rate in each sleep stage	Daily	The average breathing rate in each sleep stage (awake, light, REM, deep).
		SD breathing rate	Daily	Standard deviation of breathing rate
		Approximate entropy of BBI	Daily	The approximate entropy of the breath-to-breath intervals.

AVG, average; SD, standard deviation; BBI, beat-to-beat interval; ENMO, Euclidian Norm Minus One.

Our feature extraction engine processes ActiGraph's data to extract physical activity features. It also processed Emerald's breathing and sleep stage signals to extract meaningful features of breathing and sleep patterns. A representative feature set is presented in Table 1. All features are extracted on a daily basis with a day starting at 12pm and ending at 11:59 am the following day for activity features and starting at 7pm and ending at 6:59pm in the following day for breathing and sleep features. The day definition for breathing and sleep was chosen around sleep time to ensure sleep and breathing features from the same night are not split across two different days.

### 2.4 Measures

#### 2.4.1 Clinical assessments and participant-reported measures

##### 2.4.1.1 Self-reported eDiary

Science 37's Network Oriented Research Assistant (NORA) (19), a custom cloud-based web and mobile application, was provided to participants for completion of an electronic symptom diary (eDiary). eDiary daily measurements include fatigue severity, pain severity, emotional health, cognitive clarity, ability to perform activities of daily living, sleep satisfaction, and feeling depressed (see Table 1 for more details). The eDiary measures used in this study were custom scales using a vertical bar and a slider that could be used to provide the rating on a 0–100 scale. The numeric score was also displayed on the screen and a grey line on the scale represented the previous rating provided as a reference. While the scales used in this study were custom scales, similar 0–100 VAS scales were previously used and validated in SLE and SjD literature to monitor symptoms such as fatigue, sleep and pain (29, 30).

##### 2.4.1.2 Disease activity

During screening and at the end of the study, SLE and SjD participants underwent clinical evaluations, including measures of disease activity, completed by trained personnel in participants’ home. For SLE, we used the Systemic Lupus Erythematosus Disease Activity Index 2000 (SLEDAI-2k) (21), a measure widely used by clinicians to measure SLE activity and it ranges between 0 and 105 indicating no activity (0), mild (1–5), moderate (6–10), severe (11–19), and very severe activity (+20). Note that mobile phlebotomy was performed by mobile nurses during the in-home screening visit to obtain the laboratory values required for SLEDAI (Ig Isotype profile, C3 and C4 complement, Anti-dsDNA).For SjD, we used the EULAR Sjögren's Disease Activity Index (ESSDAI) (20), another widely used measure for SjD activity scoring with scores ranging from 0 to 36 indicating low (0–3), moderate (4–8) and high (9–36) disease activity. Required laboratory measures were collected from medical records, when available, and used by a trained rheumatologist/internist to adjudicate the ESSDAI.

#### 2.4.2 Digital health measures

##### 2.4.2.1 Physical activity features

Physical activity features are extracted from the Actigraph device. Days with less than 10 h of wear time were marked as invalid and excluded from the analysis. The raw tri-axial accelerometer data collected in the study were used to derive multiple endpoints representing participants' mobility patterns. A summary of the extracted features is provided in Table 1.

The accelerometer data was processed as follows:

1. Time gap imputation: time gaps in the data were imputed as zeros and considered as non-wear.
2. Auto-calibration: non-movement periods were removed to calculate the calibration error in each axis using the first day of data and then the auto-calibration step was applied to the signals to correct the calibration error as a pre-processing step (31).
3. Calculation of acceleration metrics:
  - ENMO (Euclidean Norm Minus One with negative values rounded to zero):
    $$ENMO=\sqrt{acc_{x}^{2}+acc_{y}^{2}+acc_{z}^{2}}-1\;(if\;ENMO\;<0,\;ENMO\;=0)$$
4. Measure rhythm features characterizing daily mobility patterns. The features we extracted are inspired by previous research (32, 33) and are defined as follows:
  - Average acceleration in each day.
  - Start time of most active 10 h of the day (M10).
  - Start time of least active 5 h of the day (L5).
  - The relative amplitude (RA) reflects the difference in activity level between the 10 most active (M10) and five least active hours (L5) in the day. Higher RA indicates a more robust 24-hour rest-activity pattern (32, 33) and is measured as follows:$RA=\frac{M10\;-\;L5}{M10\;+\;L5}$
  - Intra-daily variability (IV) which represents the relative fragmentation within days based on how many transitions occur between activity and rest. Higher IV is a measurement of fragmentation of the rest-activity pattern. IV is measured as follows:
    $$IV=\frac{n\sum_{i=2}^{n}\;{\left(x_{i}-x_{i-1}\right)}^{2}}{(n-1)\sum_{i=1}^{n}\;{\left(x_{i}-\bar{x}\right)}^{2}}$$

Where *n* = total number of data points, $\bar{x}$ mean of all data points, $x_{i}$  = individual data points.

##### 2.4.2.2 Sleep features

The Emerald system processes the RF signals and predicts one of four sleep stages (awake, light, REM, deep) every 30 s. Using this data, we extracted multiple features (14) to represent sleep quality as follows:

- Sleep efficiency represents the amount of time the participants spend asleep while in bed. It is calculated by dividing the amount of time spent asleep (light, REM, and deep) by the total amount of time in bed (higher values indicate better sleep quality).
- The time spent in every sleep stage [i.e., awake, light, rapid eye movement (REM), and deep stages] in minutes per day.
- Sleep onset latency. It is the time it takes a person to fall asleep after turning the lights out. On average, a healthy person takes between 10 and 20 min to fall asleep.
- The midpoint of sleep (MSF) is the clock time between sleep onset and waking up measured in hours.
- Total sleep opportunity which is the total time awake between falling asleep and waking in minutes.
- Number of awakenings to characterize the number and duration of sleep interruptions per night.
- The complexity of sleep cycles uses sample entropy (34) and is a measure of sleep cycle regularity (16). A higher sample entropy reflects higher variability of the sleep stage signal. Our approach for measuring sample entropy is as follows: (1) we first attribute a numerical value to each sleep cycle (1 = awake, 2 = REM, 3 = light, 4 = deep), (2) create time series of sleep stages with no repetitions (e.g., a typical awake, REM, light, deep transition would look like this: 1-2-3-4 regardless of how many seconds they spend in a given stage), and finally (3) measure the sample entropy using the following formula:
  SampEn=−log(C(m+1,r)/C(m,r))

Where *m* is the length of the pattern or template (set to 1), *r* is the tolerance threshold and is set to 20% of the standard deviation (SD) of the sleep stages, C(m,r) represents the number of similar m-length template matches found in the data, and log denotes the natural logarithm.

##### 2.4.2.3 Breathing features

The Emerald device measures the chest displacement due to breathing sampled at 5 Hz. We leverage this signal to characterize breathing rate and breath-to-breath variability. Our methodology for processing the raw breathing signal is as follows:

1. We resampled the signal to 20 Hz using a spline interpolation method (35) to improve peak detection from the breathing signal,
2. We cleaned the signal by applying linear detrending followed by a fifth order 2 Hz low-pass infinite impulse response (IIR) Butterworth filter,
3. Detected peaks using the methodology presented in (36) and measured breath to breath intervals (BBI),
4. Extracted several breathing features as follows:

- The average breathing rate per day.
- Standard deviation of breathing rates.
- Average breathing rate in each sleep stage (awake, light, REM, deep).
- Approximate entropy (37) of breath-to-breath intervals (BBI) which determines the regularity of BBI series over time. Higher approximate entropy indicates higher irregularity in the BBI patterns.

### 2.5 Data analysis methods

#### 2.5.1 Change in disease activity

Change in SLEDAI and ESSDAI disease activity between screening and end of study will be evaluated to determine whether disease activity changed over the study period. The primary analysis will use paired Student's *t*-tests on the paired differences; normality of the differences were assessed for both measures using Shapiro test.

#### 2.5.2 Disease profiling

To analyze the difference between SLE, SjD and HNV participants, we average the daily features on a weekly basis for each participant and we perform a multilevel analysis of differences between cohorts using mixed effect repeated measure models (hereafter referred to as “mixed effect models”) with maximum likelihood estimation, a random intercept for participants, and random slopes for each disease cohort (38). Because age and BMI are expected to influence physical activity, sleep and breathing patterns, we added age and BMI as covariates in all models to correct for any inherent effect of those two variables. We correct for multiple comparisons (SLE vs. HNV, SjD vs. HNV) using the Bonferroni correction method for each feature independently (39). Effect size is estimated using Cohen's d method (40). As reported in the literature, 0.2, 0.50, and 0.8 are used as thresholds to interpret small, medium, and large effect (41). Results will be reported in the following format (B, p, d) with B denoting the coefficient estimate of the fixed effects, p indicating the *p*-value associated with each estimate, and d representing the Cohen' d effect size.

#### 2.5.3 Correlation between the digital measures and self-reported symptoms

We first aggregate the self-reported eDiary measures and the daily digital measures on a weekly basis. This is motivated by the fact that participants can respond to the eDiary any time during the day which makes the definition of the past 24 h different over time. Then, because participants have multiple weekly data pairs of digital measures and self-reported symptoms, we report repeated measure correlations (42) to describe the association between the digital measures and the eDiary data.

#### 2.5.4 Correlation between the digital measures and disease activity

We use spearman correlation to measure the association between the digital measures and SLE and SjD disease activity. We average the digital measures in the last 30 days of the study (to correspond to the recall period of some items in SLEDAI and ESSDAI) and correlate them with SLEDAI and ESSDAI.

## 3 Results

### 3.1 Demographic and baseline characteristics

From the 99 enrolled participants, 4 participants discontinued the study procedures resulting in an analysis set of 95 participants. The participants in this decentralized study were enrolled across 11 states spanning all major geographic regions of the United States including the Northeast, Midwest, South, Southwest, and West. The demographic and baseline characteristics for participants included in this analysis (a total of 95 participants; 29 participants in SLE cohort, 29 participants in SjD cohort, and 37 participants in HNV cohort) are summarized in Table 2. Most of the participants were females with only 1 male participant enrolled in the SLE cohort of the study. The median age of participants was 51 years in SLE cohort, 54 years in SjD cohort, and 51 years in HNV cohort. 2 participants in SLE cohort, 3 participants in SjD cohort, and 9 participants in HNV cohort, were of Hispanic or Latino ethnicity. The ethnicity of 1 participant in the SLE cohort was not reported.

**Table 2:** Summary of demographics at baseline.

	Cohorts		
	SLE	SjD	HNV
N	29	29	37
Sex			
Male	1 (3.4%)	0	0
Female	28 (96.6%)	29 (100.0%)	37 (100.0%)
Age			
Mean(SD)	48.97 (9.39)	52.10 (10.55)	48.24 (11.32)
Median	51.00	54.00	51.00
IQR	(42.00; 55.00)	(48.00; 60.00)	(38.00; 57.00)
Range	(27.0; 63.0)	(26.0; 65.0)	(26.0; 64.0)
Ethnicity			
Hispanic or Latino	2 (6.9%)	3 (10.3%)	9 (24.3%)
Not Hispanic or Latino	26 (89.7%)	26 (89.7%)	28 (75.7%)
Not reported	1 (3.4%)	0	0
Race			
White	15 (51.7%)	24 (82.8%)	18 (48.6%)
Asian	1 (3.4%)	1 (3.4%)	9 (24.3%)
Multiple	1 (3.4%)	1 (3.4%)	1 (2.7%)
Note Reported	0	0	3 (8.1%)
Black or African American	12 (41.4%)	3 (10.3%)	6 (16.2%)
Weight (kg)			
Mean(SD)	73.90 (13.74)	71.07 (10.25)	69.33 (12.46)
Median	73.00	70.00	68.50
IQR	(68.00; 83.00)	(65.00; 76.00)	(60.00; 79.00)
Range	(48.0; 98.0)	(55.0; 92.0)	(46.0; 94.0)
BMI (kg/m^2^)			
Mean(SD)	26.37 (3.53)	26.02 (3.27)	25.97 (3.66)
Median	25.90	25.78	26.38
IQR	(23.59; 29.06)	(23.53; 28.67)	(22.95; 28.69)
Range	(19.5; 32.2)	(19.7; 32.3)	(19.2; 31.8)
SLEDAI at Baseline			
Mean(SD)	9.76 (3.55)	-	-
Median	9	-	-
IQR	(8;11)	-	-
SLEDAI Domains			
Central Nervous System	3 (10.34%)	-	-
Constitutional	1 (3.44%)	-	-
Hematologic	7 (24.13%)	-	-
Immunologic	9 (31.03%)	-	-
Mucocutaneous	26 (89.65%)	-	-
Musculoskeletal	18 (62.06%)	-	-
Renal	9 (31.03%)	-	-
Serosal	7 (24.13%)	-	-
Vascular	0 (0%)	-	-
ESSDAI at Baseline			
Mean(SD)	-	9.66 (6.83)	-
Median	-	9	-
IQR		(4;13)	
ESSDAI Domains			
Articular	-	19 (65%)	-
Biological	-	1 (3%)	-
Central Nervous System	-	0 (0%)	-
Constitutional	-	12 (41.37%)	-
Cutaneous	-	4 (13.79%)	-
Glandular	-	9 (31.03%)	-
Haematological	-	2 (6.89%)	-
Lymphadenopathy	-	12 (41.37%)	-
Muscular	-	0 (0%)	-
Peripheral Nervous System	-	12 (41.37%)	-
Pulmonary	-	4 (13.79%)	-
Renal	-	0 (0%)	-
Time Since Diagnosis (years)			
Mean (SD)	12.3 (9.58)	10.3 (10.3)	-
Median	11	6	-
IQR	(2.5; 20.5)	(3.5; 13)	-
Concomitant Medication			
Antimalarials	22 (75.86%)	14 (48.27)	-
Immunosuppressants	14 (48.27)	3 (10.34%)	-
Glucocorticoids	14 (48.27)	5 (17.24)	-

SD, standard deviation; IQR, interquartile range.

The majority of the enrolled participants were White (15 participants in SLE cohort, 24 participants in SjD cohort, and 18 participants in HNV cohort) followed by Black or African American participants (12 participants in SLE cohort, 3 participants in SjD cohorts, and 6 in HNV cohort). The median body mass index (BMI) of participants was 25.90 kg/m2 in SLE cohort, 25.78 kg/m2 in SjD cohort, and 26.38 kg/m2 in HNV cohort.

At baseline, the SLEDAI total mean score (SD) within the SLE cohort was 9.76 ± 3.552. On the other hand, the ESSDAI total mean score (SD) within the SjD cohort was 9.66 ± 6.831 at baseline.

Given the previous information, the baseline demography of participants was similar across the cohorts with respect to age, race, ethnicity and BMI.

### 3.2 Data coverage

The median average wear time per 24 h of CPIW during the study duration was 21.8 h for SLE cohort, 21.8 h for SjD cohort, and 21.9 h for HNV cohort corresponding to (90% in SLE, 90% in SjD, and 91% in HNV). The missing actigraphy data resulted in 600 (5.12%) nights excluded from the comparison with daily PROs.

Coverage of Emerald device was defined as number of days in which sleep and breathing data was recorded divided by the number of days in which we expect data. The overall coverage is measured by averaging the coverage of each participant. Overall, the coverage in 69 participants is high (91% in SLE, 93% in SjD, 88% in HNV). The missing Emerald data resulted in 721 (9.02%) nights excluded from the comparison with daily PROs.

**Figure 4:**
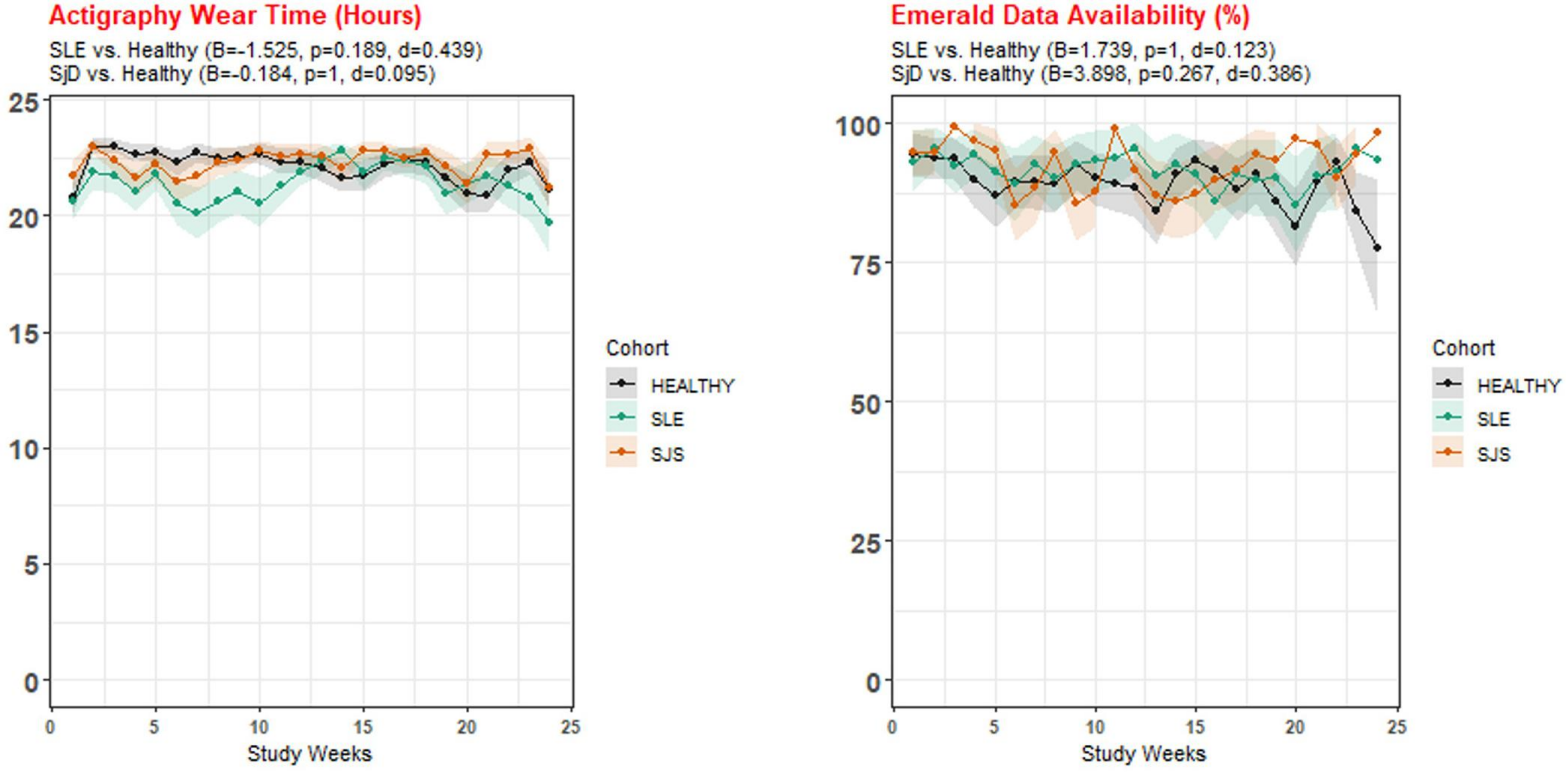
Data coverage over time across the two digital devices.

As presented in Figure 4, the data coverage from both devices is consistent over time and across the three groups.

**Figure 5:**
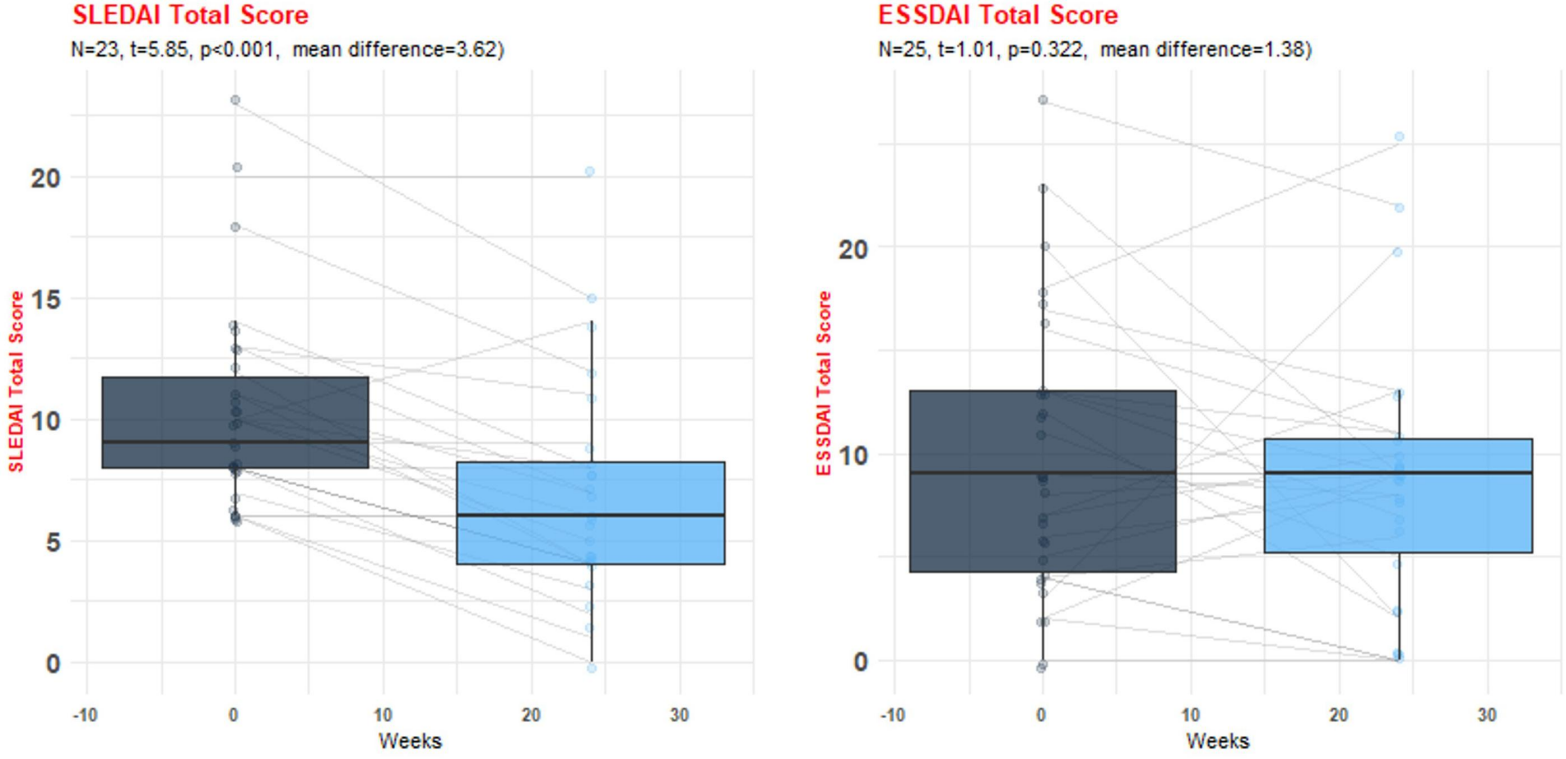
Change in SLEDAI and ESSDAI disease activity scores from baseline (week 0) to the end of the study (week 24).

### 3.3 Disease profiling

#### 3.3.1 Change in disease activity

As presented in Figure 5, there was a modest but significant difference between SLEDAI at baseline vs. week 24 (t = 5.85, *p* < 0.001) for SLE patients. The mean change between baseline and week 24 was 3.6 with 10 out of 23 (43%) achieving >4-point reduction in SLEDAI, which is the within-participant clinically meaningful threshold for change in SLEDAI (43). The change in SLEDAI is plausibly explained by natural disease fluctuation, measurement variability, or regression to the mean. For SjD patients, ESSDAI scores were not significantly different between baseline and week 24.

#### 3.3.2 Self-reported symptoms

**Figure 6:**
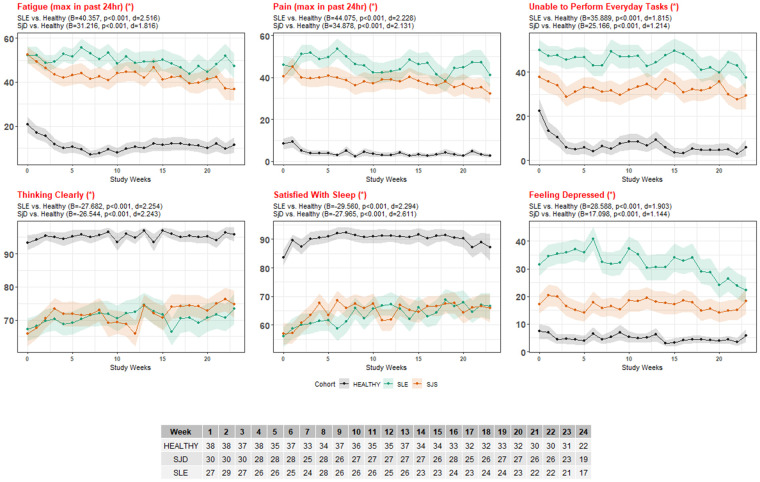
Differences in self-reported symptoms between HNV, SLE, and SjD participants. Weekly Average of Daily physical activity features. Mean (lines) and confidence intervals (ribbon) of each feature are displayed by group and over weeks of the study. Features denoted by (*) show significant differences between HNV and at least one of the two disease groups. The number of subjects in each group and in each week are displayed in the table on the bottom.

Differences in self-reported symptoms presented in Figure 6 show that SLE and SjD participants exhibited significantly worse symptoms than HNV with SLE participants having the worst symptoms. Fatigue was found significantly higher in SLE (B = 40.35, *p* < 0.001, d = 2.51), and higher in SjD (B = 31.21, *p* < 0.001, d = 1.81) than HNV. Pain was also found significantly higher in SLE (B = 44.07, *p* < 0.001, d = 2.22), and higher in SjD (B = 34.87, *p* < 0.001, d = 2.13) than HNV. SLE (B = 35.88, *p* < 0.001, d = 1.81) and SjD (B = 25.16, *p* < 0.001, d = 1.21) participants were unable to perform everyday tasks significantly more than HNV. Both SLE (B = −27.68, *p* < 0.001, d = 2.25) and SjD (B = −26.54, *p* < 0.001, d = 2.24) participants were thinking significantly less clearly than HNV. Finaly, both SLE (B = −29.56, *p* < 0.001, d = 2.29) and SjD (B = −27.96, *p* < 0.001, d = 2.61) participants were less satisfied with sleep and both SLE (B = 28.58, *p* < 0.001, d = 1.90) and SjD (B = 17.09, *p* < 0.001, d = 1.14) participants were significantly more depressed.

#### 3.3.3 Physical activity

**Figure 7:**
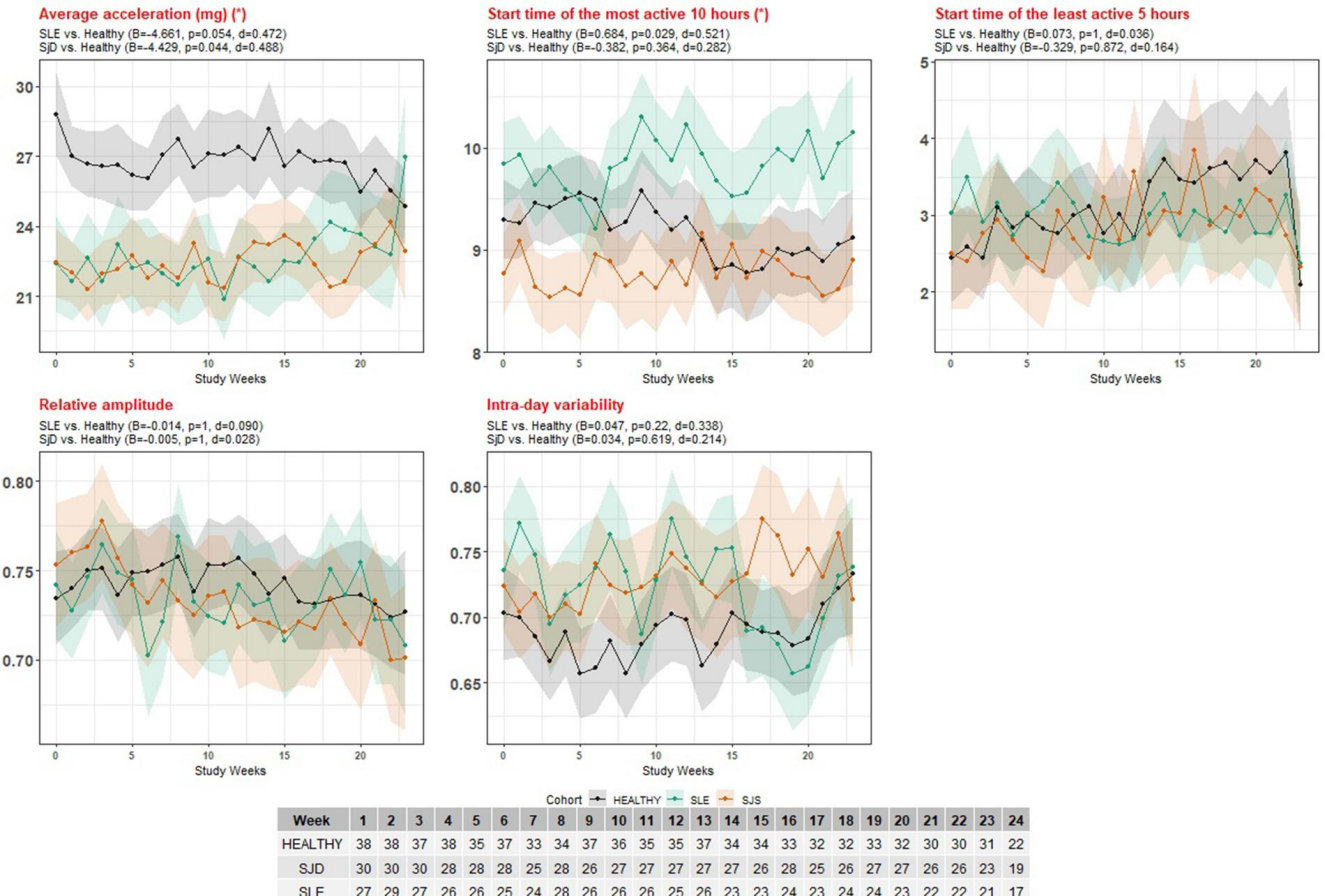
Differences in physical activity features between HNV, SLE, and SjD participants. Weekly Average of Daily physical activity features. Mean (lines) and confidence intervals (ribbon) of each feature are displayed by group and over weeks of the study. Features denoted by (*) show significant differences between HNV and at least one of the two disease groups. The number of subjects in each group and in each week are displayed in the table on the bottom.

Results of the physical activity features are presented in Figure 7. Results suggest that there is a significant difference in average daily activity level and the starting time of the most active 10 h. The average M10 starting time is 9.2 h from midnight or 9:12am in the HNV cohort, earliest (8.8 h from midnight or 8:48am) in the SJD (B = −0.38,*p* = 0.36,d = 0.28) and latest (9.9 h from midnight or 9:54am) in the SLE cohort (B = 0.68, *p* = 0.02, d = 0.52). The average acceleration is highest in the HNV cohort, significantly higher than SLE (B = −4.66, *p* = 0.05. d = 0.47) and higher than SjD (B = −4.42, *p* = 0.04, d = 0.48) suggesting that both disease groups exhibited reduction in physical activity when compared to HNV group. There are no significant differences in the start time of 5 least active hours, the relative amplitude and intra-day variability.

#### 3.3.4 Sleep

**Figure 8:**
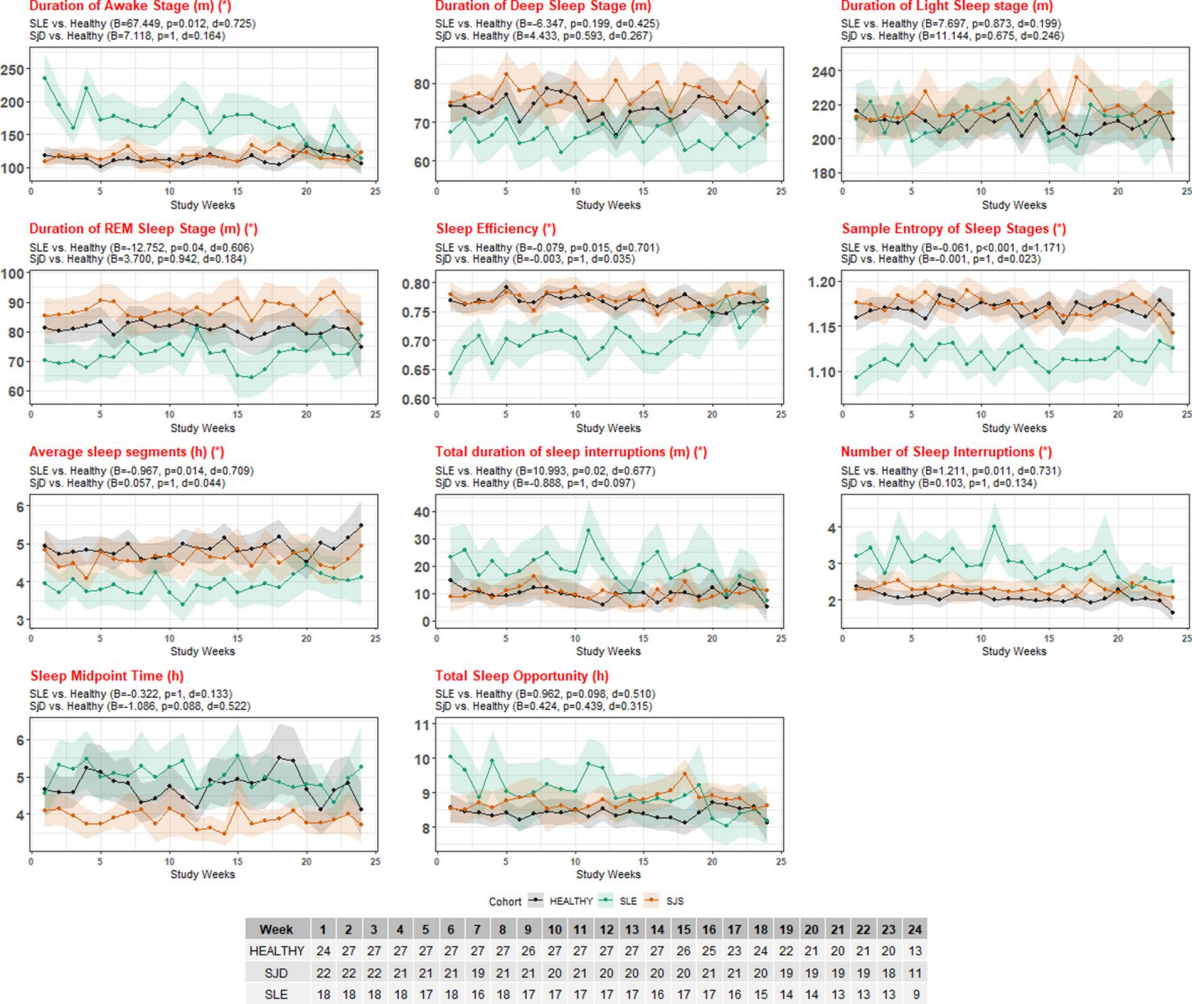
Differences in sleep features between HNV, SLE, and SjD participants. Weekly Average of Daily physical activity features. Mean (lines) and confidence intervals (ribbon) of each feature are displayed by group and over weeks of the study. Features denoted by (*) show significant differences between HNV and at least one of the two disease groups. The number of subjects in each group and in each week are displayed in the table on the bottom.

SLE participants had significantly lower sleep efficiency with a medium to large effect size (B = −0.07, *p* = 0.01, d = 0.70) with SLE participants having 7.9% lower sleep efficiency than HNV participants (see Figure 8). Looking at time spent in different sleep stages, we can observe that there is no statistical difference in time spent in light sleep. However, SLE participants were found to have more awake time in the order of 67 min higher than HNV participants (B = 67.44, *p* = 0.01, d = 0.72) and have less time in deep sleep than HNV (B = −6.34, *p* = 0.19, d = 0.42). They were also found to spend about 12 min less in REM stage (B = −12.75, *p* = 0.04, d = 0.60) per night.

The sample entropy of sleep stages reveals a significant and a large effect size (B = −0.06, *p* < 0.001, d = 1.17) with SLE participants having lower entropy than HNV participants. Lower entropy indicates less complex sleep stage trajectories. This is justified by the fact that SLE participants spent less time in the REM stage making the sleep stage traces look less complex given most of the time, the sleep is alternating between awake and light sleep instead of alternating between the four stages (see Figure 8 where we record more time in awake and light stages and less for deep and REM in SLE).

SLE participants also exhibited significantly higher sleep interruptions than the two other groups as observed by shorter sleep segments (B = −0.96, *p* = 0.01, d = 0.70), higher number of interruptions (B = 1.21, *p* = 0.01, d = 0.73), longer total duration of sleep interruption (B = 10.99, *p* = 0.02, d = 0.67). SLE participants did not show different sleep midpoint times suggesting that they tend to go to bed around the same time as HNV participants. They also did not exhibit different total sleep opportunity.

SjD participants did not show significantly different sleep patterns from HNV participants. It is worth noting that the sleep midpoint time was lower than HNV (B = −1.08, *p* = 0.08, d = 0.52) suggesting that they tend to go to bed and wake up earlier than HNV participants. This also correlates with the physical activity results measured through Actigraph and in which we recorded that SjD cohort had lower start time of 10 most active hours. Sleep midpoint time is known to be correlated with dim light melatonin onset and a reliable marker of chronotype (44). Thus, SjD participants appear to have better sleep regularity than HNV.

#### 3.3.5 Breathing

**Figure 9:**
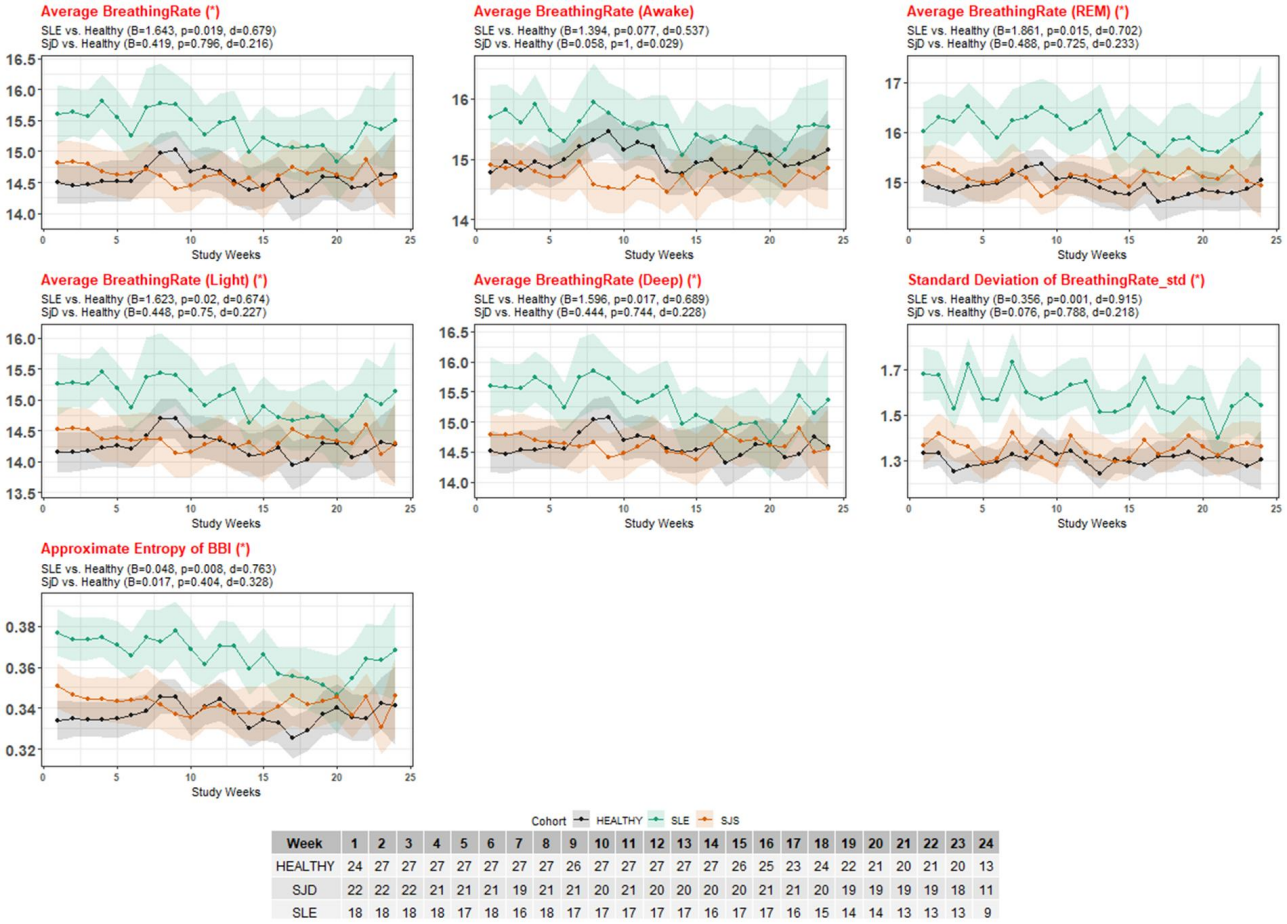
Differences in breathing features between HNV, SLE, and SjD participants. Weekly Average of Daily physical activity features. Mean (lines) and confidence intervals (ribbon) of each feature are displayed by group and over weeks of the study. Features denoted by (*) show significant differences between HNV and at least one of the two disease groups. The number of subjects in each group and in each week are displayed in the table on the bottom.

As presented in Figure 9, the average breathing rate was significantly different between SLE and HNV with SLE participants having 1.643 breaths/minute higher than HNV participants (B = 1.64, *p* = 0.01, d = 0.67) with a medium to large effect size. To investigate if breathing rate is elevated in SLE due to poor sleep quality (i.e., being more awake in the night), we measured differences in breathing rates in the four stages of sleep. Results suggest that breathing is elevated in SLE in all four stages indicating that the higher breathing rate in SLE is not mediated by poorer sleep quality (see breathing in different sleep stages in Figure 9).

Breathing variability metrics including standard deviation of breathing rate (B = 0.35, *p* = 0.001, d = 0.91) and approximate entropy of BBI (B = 0.04, *p* = 0.008, d = 0.76) were both significantly higher in SLE participants over the course of the study with a large effect size (see Figure 9), suggesting less stable breathing than the other two groups. High breathing variability was previously associated with deterioration in health quality (45, 46).

### 3.4 Correlation between the digital measures and self-reported symptoms

**Figure 10:**
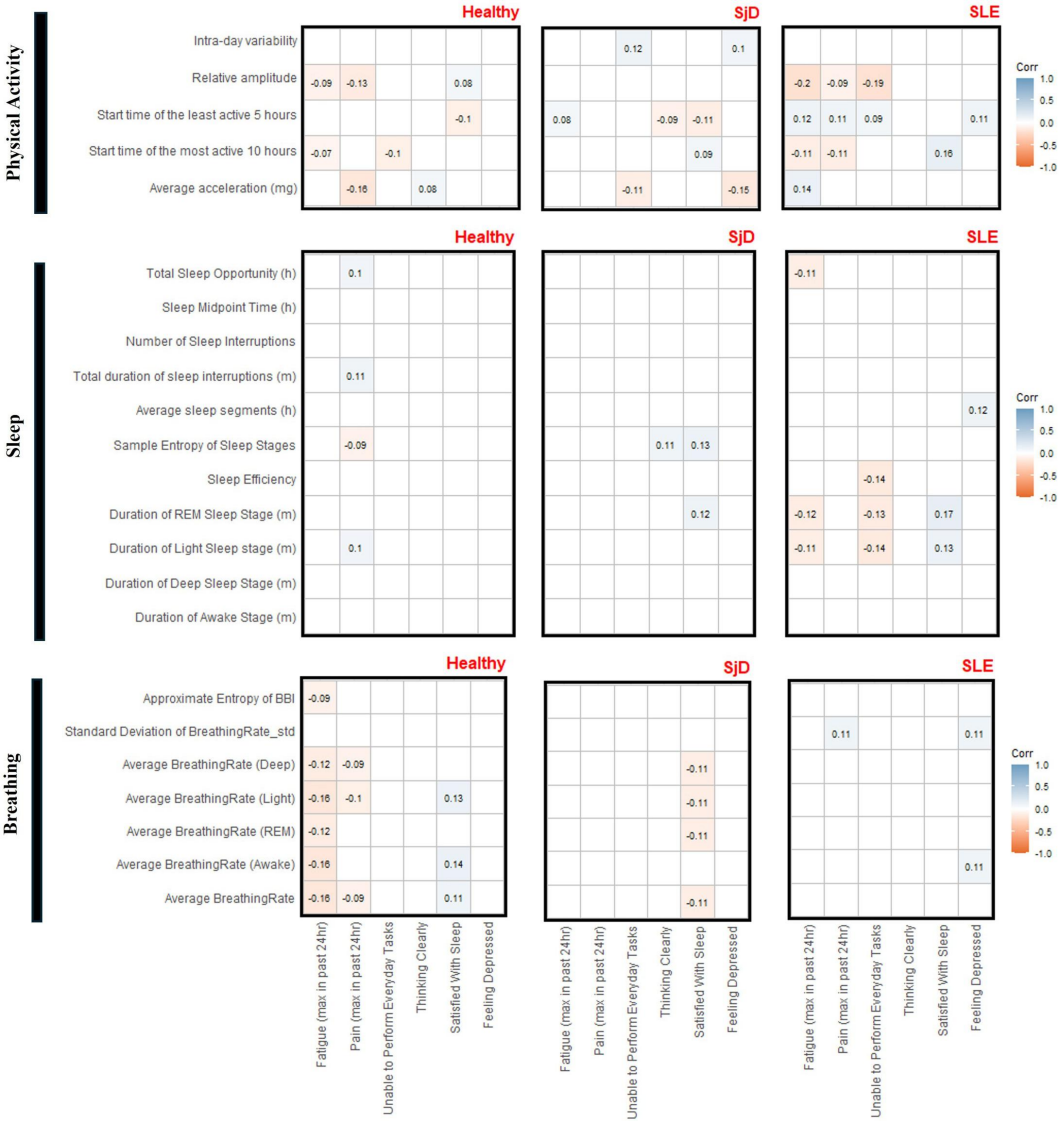
Repeated correlation between digital measures and self-reported measures. Only significant (*p* < 0.05) correlations are displayed.

Repeated measured correlations between the digital measures and the self-reported measures are displayed in Figure 10. Results are masked to display only significant correlations (i.e., *p*-value < 0.05). Overall, the measures were weakly correlated with coefficients ranging between 0.08 and 0.19. Correlation patterns are also varying by cohorts, may be due to small sample size or to actual differences in the types of digital measures associated with the concepts, but overall correlations are in the expected direction. For instance, in both SLE and SjD (but not HNV), fatigue is weakly positively correlated with the L5 (a later time of least active hours is associated with greater fatigue). Fatigue was also negatively correlated with breathing in HNV indicating reduction in fatigue is associated with higher breathing rates. The strongest within-participant correlations observed are on the order of 0.2 in the relative amplitude of the SLE cohort. This indicates that in weeks where the average amplitude was larger for the circadian rhythm, the fatigue was reported to be lower, and the participants were able to do their daily tasks. Additionally, in both SLE and SjD (but not HNV), sleep satisfaction is correlated with a longer duration of REM sleep. Sleep satisfaction is also positively associated with breathing rate during sleep in HNV but negatively in SjD.

### 3.5 Correlation between the digital measures and disease activity

Given that differences were identified in self-reported symptoms and the digital health measures between both the SLE and SjD participants vs. HNV volunteers, we sought to understand whether these differences were associated with disease activity. The spearman correlations between the eDiary, physical activity, sleep and breathing measures and SLE and SjD disease activity are displayed in Table 3. Note that the number of participants included in this analysis is displayed on the table and is different across the different features and between the two disease groups because (1) we had more participants with Actigraph (i.e., physical activity measures) than participants with Emerald (i.e., sleep and breathing measures), and (2) some participants did not complete the end of the study SLEDAI or ESSDAI assessments and were excluded from the correlation analysis.

**Table 3:** Spearman correlation between the digital measures of physical activity, sleep and breathing with disease activity.

	Correlation with SLEDAI			Correlation with ESSDAI		
	N	r	*p*-value	N	r	*p*-value
Self-reported symptoms						
Fatigue	24	0.01	0.96	26	0.35	0.08
Pain	24	0.17	0.43	26	0.14	0.49
Ability to perform daily tasks	24	0.01	0.94	26	−0.19	0.36
Cognitive health	24	−0.16	0.46	26	−0.22	0.29
Sleep satisfaction	24	**−0**.**45**	**0**.**03**	26	−0.22	0.28
Depression	24	−0.30	0.16	26	−0.25	0.21
Physical Activity	24	0.01	0.96	26	0.35	0.08
Average acceleration	23	−0.23	0.29	26	0.05	0.80
Start time of the 10 most active hours	23	0.22	0.32	26	0.11	0.60
Start time of the 5 least active hours	23	0.25	0.25	26	0.04	0.85
Intra-daily variability	23	0.04	0.84	26	0.01	0.96
Relative amplitude	23	0.38	0.08	26	0.04	0.86
Sleep						
Duration of being awake	16	0.08	0.77	21	−0.01	0.95
Duration of deep sleep	16	0.21	0.44	21	−0.22	0.33
Duration of light sleep	16	0.00	1.00	21	0.12	0.62
Duration of REM sleep	16	−0.02	0.93	21	−0.04	0.87
Sleep efficiency	16	−0.09	0.73	21	0.03	0.89
Entropy of sleep stages	16	−0.17	0.54	21	−0.34	0.13
Average sleep segments	16	−0.01	0.97	21	−0.01	0.96
Total duration of sleep interruptions	16	0.25	0.35	21	0.26	0.26
Number of Sleep Interruptions	16	0.25	0.35	21	−0.06	0.81
Sleep Midpoint Time	16	0.12	0.67	21	−0.06	0.8
Total Sleep Opportunity	16	0.09	0.74	21	0.11	0.63
Breathing						
AVG breathing rate	16	0.41	0.11	21	−0.08	0.73
AVG breathing when awake	16	**0**.**50**	**0**.**05**	21	0.06	0.81
AVG breathing during light sleep	16	0.40	0.12	21	−0.17	0.47
AVG breathing during REM sleep	16	0.45	0.08	21	−0.07	0.75
AVG breathing during deep sleep	16	0.42	0.11	21	−0.05	0.84
SD breathing rate	16	**0**.**56**	**0**.**02**	21	0.24	0.29
Approximate entropy of BBI	16	0.48	0.06	21	−0.02	0.94

Bold values represent statistically significant correlations (i.e., *p*-value<0.05).

**Figure 11:**
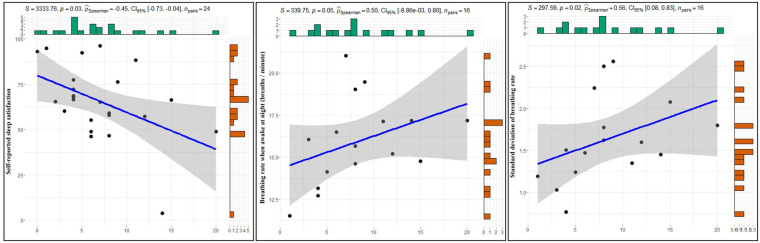
Correlation between self-reported sleep satisfaction, breathing rate when awake, and standard deviation of breathing rate with SLEDAI.

Results suggest that from the self-reported measures, only sleep satisfaction correlated with SLEDAI (r = −0.45, *p* = 0.03) indicating that SLE patients with greater disease activity generally reported lower sleep satisfaction. From the digital measures, higher breathing rate, [especially when awake at night (r = 0.50, *p* = 0.05)], in addition to higher breathing variability (as captured by standard deviation of breathing rate (r = 0.56, *p* = 0.02) are associated with higher SLE disease activity. Scatter plots of the three measures with statistically significant correlations with SLEDAI are displayed in Figure 11.

Because the disease activity measures are composite scores that group multiple domains and organ systems, analyzing only total composite measures may mask subtle but significant differences. Thus, we have included the correlation between individual assessment items from SLEDAI-2k and ESSDAI and the objective sensor data in the Supplementary Materials. For instance, it was found that the artheratis item form SLEDAI-2k is negatively correlated with average acceleration and sleep efficiency, and positivity correlated with sleep interruptions and amount of time spent awake at night.

On the other hand, none of the features correlated strongly with ESSDAI.

## 4 Discussion

This study employed an innovative virtual design leveraging Science 37's Network Oriented Research Assistant (NORA) platform, enabling seamless communication between participants and study personnel and an integrated ecosystem of medical support. Consequently, we monitored 95participants over an extended 6-month period, yielding one of the richest multimodal datasets described to date in SLE and SjD. Although cohort sizes were modest (SLE *n* = 29, SjD *n* = 29, healthy controls *n* = 37), the sample sizes were guided by ensuring sufficient power for making between-cohort comparisons using assumptions from the literature from smaller studies. The combination of continuous sleep and physical activity measurements made across a geographically diverse US sample represents a key strength and novel contribution of this work.

### 4.1 Principal results

SLE and SjD participants exhibited significantly worse symptoms than HNV with SLE participants having more severe symptoms. SLE and SjD had significantly more pain, fatigue, and depression, had significantly less satisfaction with sleep, less ability to perform daily activities and were thinking clearly less than HNVs.

Results derived from Actigraph and Emerald data suggest that many digital measures differentiate SLE participants from demographic-matched HNV participants. SLE participants exhibited lower physical activity during the day and a peak activity time coming later than HNV participants. Furthermore, they were found to have significantly less efficient sleep, less REM sleep, more awake time during the night and exhibited more interrupted sleep indicating poorer sleep quality than HNV participants. They also showed higher breathing rates and more variability in breath-to-breath intervals. This potentially suggests that breathing, mobility, and sleep are all dysregulated in SLE and demonstrates the importance of multi-modal sensing at capturing multiple dimensions of disease state. Correlation with SLE disease activity indicated that higher breathing rate and higher breathing variability were associated with more severe disease activity. Some individual domains from the disease activity score such as the presence of Arthritis were also correlated with reduced physical activity and poorer sleep quality. These findings provide preliminary evidence of the utility of using passive sensing and non-contact sensing to monitor symptoms of SLE. SjD participants showed a reduction in physical activity (similarly to SLE patients) with an earlier peak activity time. However, no differences were found in sleep and breathing. One potential hypothesis for this is that the SjD patient population enrolled in this study was milder in terms of disease activity than the SLE patient population. An alternative hypothesis is that differences in breathing rate and disrupted sleep are more common in SLE than SjD patients. Given that these are both systemic and heterogenous diseases, future work should seek to test these hypotheses by evaluating breathing rate during sleep in a larger sample of patients.

Across individual disease-activity items, passively captured digital features (physical activity, sleep, breathing) that are presented in Supplementary Appendix 3, showed generally small cross-sectional correlations, with few statistically significant associations—consistent with evidence that daily-life behavior and physiology reflect multidimensional influences (fatigue, sleep quality, pain, mood, deconditioning, medications) that only partially overlap with clinician-scored items. Directionally plausible patterns emerged: articular/musculoskeletal involvement tended to align with lower activity and more fragmented rest–activity rhythms, whereas mucocutaneous items showed limited coupling to digital features. Serosal involvement may plausibly impact breathing and sleep due to chest discomfort. Hematologic items exhibited weak alignment with digital metrics, mirroring the frequent dissociation between cytopenias and day-to-day symptom burden. Low prevalence of certain items (e.g., CNS, renal, muscular) constrained power for those domains.

Baseline correlations between digital measures and laboratory indices, presented in SupplementaryAppendix 4, were likewise small, supporting the view that these modalities provide complementary—not redundant—information about disease impact in systemic autoimmunity. A focused set of biologically plausible associations included: fatigue with hemoglobin/hematocrit; later timing of peak daily activity with lower lymphocyte counts; longer light-sleep duration with higher C3; lower sleep satisfaction with higher platelets; and higher average respiratory rate (overall, awake, REM) with higher lymphocyte counts, while reduced beat-to-breath interval complexity related to lower red blood cell counts.

Overall, because correlations between passive digital-sensing measures and established patient-reported outcomes were weak across both SLE and SjD, passive sensing does not appear to substitute for clinical assessment or PROs. However, it may offer objective, longitudinal data capturing different aspects of patients' status and could therefore augment—rather than replace—traditional measures.

### 4.2 Limitations

Although this work is one of the first works investigating the use of passive sensing to continuously monitor physical activity, breathing, and sleep together with patient-reported symptoms of SLE and SjD in patients' homes, it still suffers from few limitations. One of the limitations of the study design is that the digital measures could not be measured during the recall period of the initial disease assessments at the start of the study. As such, the correlation between within-patient change in the digital measures and changes in disease activity measures was not possible. Moreover, the small sample size led to lower power to detect small to medium sized effects especially when measuring correlations with disease activity. Finally, while the clinical assessments including SLEDAI and ESSDAI were captured by highly trained nurses, these assessments were not performed by rheumatologists and were captured in the home environment which may introduce additional measurement variability into the disease activity scores. Additionally, for SjD participants, the lab measures needed for the biological domain of ESSDAI were captured from medical records. This approach was tailored to a remote, non-interventional study but precluded the use of some disease-specific lab values for correlation analyses with digital measures.

While we have corrected for multiple comparisons (SLE vs. HNV, SjD vs. HNV) using the Bonferroni correction method, this correction was performed for each feature independently and cross-features adjustment was not performed. Given the exploratory, hypothesis-generating nature of these analyses, the adjusted *p*-values should be interpreted as a guide rather than confirmatory evidence.

Participants were overwhelmingly female, aligning with previously reported female:male ratios of about 9:1 in SLE (47) and 9–11:1 in SjD (48). Given that only one male was enrolled, and that sex differences exist in respiratory physiology, sleep, and patterns of physical activity, our results should be interpreted with caution when applied to male patients.

Additionally, we did not specifically study the impacts of the COVID-19 pandemic on the signals collected in this study. The study enrollment paused for 6 months during the height of the COVID-19 pandemic to protect study participants. The study pause was due to the in-home nurse visit required at the start of the study. Participants already enrolled in the study during the pause continued collection and had the option to delay any upcoming in-home visits while still continuing with remote monitoring. 29 participants were enrolled before the pause and 70 after the pause. However, because recruitment used a dynamic matching design that enrolled all cohorts concurrently over the 2019–2022 period and data collection occurred within overlapping time frames across groups, any pandemic-related disruptions are unlikely to differentially bias between-group comparisons. Furthermore, the Emerald data was collected only after the pause in enrollment due to the COVID-19 pandemic. Thus, we anticipate only minimal impact of COVID-19 on the study's main outcomes. Nevertheless, we acknowledge that broader population-level changes due to the pandemic could still influence habitual activity or sleep, and we did not model these temporally.

### 4.3 Comparison with prior work

The findings discussed in this paper align with established symptomology of SLE—fatigue, fever, pain, and swelling—that substantially impair health-related quality of life and are frequently associated with reduced physical function and mobility, poorer sleep quality, and disordered nocturnal breathing (49–52). Multiple patient-reported outcomes and clinic-based cohorts demonstrate that poorer musculoskeletal activity (e.g., due to active arthritis) and higher symptom burden correlate with decrements in physical functioning and mobility domains, as well as greater fatigue and pain interference in SLE (53, 54). Our observations are also concordant with polysomnography-based evidence in SLE showing objective sleep disruption—elevated apnea–hypopnea indices, sleep fragmentation, reduced sleep efficiency, and periodic limb movements—indicative of impaired sleep quality and nocturnal respiratory/movement disorders (52, 55–58). In parallel, literature in SjD similarly reports a high prevalence of insomnia, excessive daytime sleepiness, and elevated risk for sleep apnea, with sleep disturbances strongly linked to fatigue and reduced quality of life—supporting a mechanistic pathway by which disease activity and symptom burden contribute to decreased functional capacity and mobility (59). Taken together, prior work in SLE and SjD are in line with the findings of this work and indicate that higher disease activity and symptom burden are associated with reductions in mobility and physical functioning and with poorer sleep quality; objective nocturnal studies (including polysomnography) frequently reveal respiratory and movement abnormalities that further reinforce the clinical signal of compromised sleep in these conditions (52, 60, 61).

## 5 Conclusions

This paper discussed the use of passive sensing to extract physical and physiological signals that can characterize symptoms of autoimmune diseases. We presented a set of features that represent physical activity, sleep and breathing and explored how they differ between SLE, SjD and HNV participants in a 6-months in-home study including 29 SLE, 29 SjD and 37 HNV participants. Results show increased self-reported fatigue, pain, and depression and decreased ability to perform daily tasks, satisfaction with sleep and thinking clearly in SLE and SjD when compared to HNV participants. Results derived from objective digital measures also show that physical activity is dysregulated in both SLE and SjD and that sleep and breathing are significantly dysregulated in SLE. This provides preliminary evidence of the importance of using passive sensing to understand quality of life for patients living with autoimmune diseases.

## Data Availability

The datasets presented in this article are not readily available because of the data-sharing policy of Johnson & Johnson. Requests for access to the study data can be submitted through the Yale Open Data Access (YODA) Project site. Requests to access the datasets should be directed to https://yoda.yale.edu/.
