# Supplementary material for: Using objective measures of physical activity, sleep, and breathing for disease profiling of patients with systemic lupus erythematosus and Sjögren's disease
Source: Front Digit Health. 2026 May 29;8:1759967. doi: 10.3389/fdgth.2026.1759967 (PMC13260165; doi:10.3389/fdgth.2026.1759967)
Supplement: Supplementary file 1 [file Supplementaryfile1.docx]

Using Objective Measures of Physical Activity, Sleep, and Breathing for Disease Profiling of Patients with Systemic Lupus Erythematosus (SLE) and Sjögren’s Disease (SjD)

Mehdi Boukhechba^1^, Zhi Li^1^, Elena Reynoso^1^, Ioannis Pandis^1^, Kenneth Mosca^1^, Mark Morris^1^, Stefan Avey^1^

^1^Johnson & Johnson, New Brunswick, NJ, United States

*** Correspondence:**Mehdi Boukhechba
[mboukhec@its.jnj.com](mailto:mboukhec@its.jnj.com)

# Appendix 1: Additional Medication Specifications

Table 1: Concomitant Glucocorticoids (GC) dosage.

| **Glucocorticoids** | **Frequency** | | | | | | | | | | |  |
| --- | --- | --- | --- | --- | --- | --- | --- | --- | --- | --- | --- | --- |
|  | **ONCE** | | **QD** | | **BID** | | **PRN** | | **Other** | | |  |
|  | **N** | **MD** | **N** | **MD** | **N** | **MD** | **N** | **MD** | | **N** | **MD** | |
| **Original dosage** | | | | | | | | | | | |  |
| **SjD** | | | | | | | | | | | |  |
| **FLUDROCORTISONEACETATE** | **0** | 0 | **0** | 0 | **1** | 0.1 | **0** | 0 | | **0** | 0 | |
| **METHYLPREDNISOLONE** | **0** | 0 | **0** | 0 | **0** | 0 | **0** | 0 | | **1** | 4 | |
| **PREDNISONE** | **0** | 0 | **1** | 10 | **0** | 0 | **2** | 7.5 | | **0** | 0 | |
| **SLE** | | | | | | | | | | | |  |
| **DEXAMETHASONE** | **0** | 0 | **0** | 0 | **0** | 0 | **1** | 5 | | **0** | 0 | |
| **PREDNISONE** | **0** | 0 | **9** | 5.22 | **2** | 3.5 | **2** | 10 | | **1** | 2.5 | |
| **TRIAMCINOLONEACETONIDE** | **1** | 40 | **0** | 0 | **0** | 0 | **0** | 0 | | **0** | 0 | |
| **Prednisone-equivalent dosage** | | | | | | | | | | | | |
| **SjD** | | | | | | | | | | | | |
| **FLUDROCORTISONEACETATE** | **0** | 0 | **0** | 0 | **1** | N/A | **0** | 0 | | **0** | 0 | |
| **METHYLPREDNISOLONE** | **0** | 0 | **0** | 0 | **0** | 0 | **0** | 0 | | **1** | 5.0 | |
| **PREDNISONE** | **0** | 0 | **1** | 10 | **0** | 0 | **2** | 7.5 | | **0** | 0 | |
| **SLE** | | | | | | | | | | | | |
| **DEXAMETHASONE** | **0** | 0 | **0** | 0 | **0** | 0 | **1** | 33.3 | | **0** | 0 | |
| **PREDNISONE** | **0** | 0 | **9** | 5.22 | **2** | 3.5 | **2** | 10 | | **1** | 2.5 | |
| **TRIAMCINOLONEACETONIDE** | **1** | 50 | **0** | 0 | **0** | 0 | **0** | 0 | | **0** | 0 | |
| - QD: Once a day, BID: Twice a day, PRN: As needed: MD: Mean Dosage in mg - Note that there is one SLE subject on two glucocorticoids (TRIAMCINOLONEACETONIDE and PREDNISONE) - Prednisone-equivalent dose (mg) = original dose (mg) × conversion factor. Conversion factors (anti-inflammatory equivalence)^1–3^:   - Prednisone: 1.00   - Methylprednisolone: 5/4=1.25   - Triamcinolone acetonide: 5/4=1.25   - Dexamethasone: 5/0.75≈6.67   - Fludrocortisone acetate: not routinely converted to prednisone-equivalent because of predominant mineralocorticoid activity | | | | | | | | | | | | |

Table 2: Detailed specification of Concomitant Immunosuppressants

| **Type of Immunosuppressants** | **Subjects** |
| --- | --- |
| **SJD** | |
| **AZATHIOPRINE** | 1 |
| **BELIMUMAB** | 0 |
| **LEFLUNOMIDE** | 1 |
| **METHOTREXATE** | 1 |
| **MYCOPHENOLATE MOFETIL** | 0 |
| **SLE** | |
| **AZATHIOPRINE** | 3 |
| **BELIMUMAB** | 4 |
| **LEFLUNOMIDE** | 1 |
| **METHOTREXATE** | 1 |
| **MYCOPHENOLATE MOFETIL** | 8 |

Table 3: The distribution of SLEDAI-2K items.

| **SLEDAI-2k Items** | **SLE subjects** |
| --- | --- |
| **Alopecia** | 21 (72.41%) |
| **Rash** | 21 (72.41%) |
| **Arthritis** | 18 (62.07%) |
| **Mucosal Ulcers** | 14 (48.28%) |
| **Low complement** | 9 (31.03%) |
| **Pleurisy** | 6 (20.69%) |
| **Proteinuria** | 6 (20.69%) |
| **Leukopenia** | 5 (17.24%) |
| **Increased DNA binding** | 4 (13.79%) |
| **Hematuria** | 3 (10.34%) |
| **Myositis** | 2 (6.90%) |
| **Pericarditis** | 2 (6.90%) |
| **Thrombocytopenia** | 2 (6.90%) |
| **Fever** | 1 (3.45%) |
| **Lupus Headache** | 1 (3.45%) |
| **Organic Brain Syndrome** | 1 (3.45%) |
| **Pyuria** | 1 (3.45%) |
| **Seizure** | 1 (3.45%) |
| **Cerebrovascular Accident (CVA)** | 0 (0%) |
| **Cranial Nerve Disorder** | 0 (0%) |
| **Organic Brain Syndrome** | 0 (0%) |
| **Psychosis** | 0 (0%) |
| **Seizure** | 0 (0%) |
| **Visual Disturbance** | 0 (0%) |

Table 4: Summary of laboratory results at screening

|  | **Cohorts** | | |
| --- | --- | --- | --- |
|  | **SLE** | **SjD** | **HNV** |
| **N** | 29 | 29 | 37 |
| **Complement C3 (mg/dL) (Reference: [0.9,** **1.8])** | | | |
| Mean(SD) | 1.12 (0.3) | - | - |
| Median | 1.13 | - | - |
| IQR | (0.94; 1.38) | - | - |
| **Complement C4 (mg/dL) (Reference: [0.1,** 0**.4])** | | | |
| Mean(SD) | 0.20 (0.07) | - | - |
| Median | 0.19 | - | - |
| IQR | (0.15; 0.26) | - | - |
| **Immunoglobulin G (mg/dL) (Reference: [11.6,** **16.4])** | | | |
| Mean(SD) | 12.75 (4.78) | - | - |
| Median | 12.35 | - | - |
| IQR | (9.24; 14.72) | - | - |
| **Anti-Double Stranded DNA (IU/mL)** | | | |
| Mean(SD) | 50.54 (177.59) | - | - |
| Median | 12.29 | - | - |
| IQR | (12.29; 12.29) | - | - |
| **Erythrocytes (10^12^/L) (Reference: [4.1,** **5.6])** | | | |
| Mean(SD) | 4.51 (4.07) | 3.84 (2.28) | 4 (2.05) |
| Median | 4.30 | 4.15 | 4.20 |
| IQR | (3.4; 4.7) | (3.15; 4.5) | (3.85; 4.5) |
| **Lymphocytes (10^9^/L) (Reference: [0.91,** **4.28])** | | | |
| Mean(SD) | 1.64 (0.75) | 1.83 (0.7) | 2.01 (0.62) |
| Median | 1.49 | 1.76 | 1.96 |
| IQR | (1.11;2.04) | (1.42;2.14) | (1.65;2.30) |
| **Platelets (10^9^/L) (Reference: [140,** **400])** | | | |
| Mean(SD) | 253.44 (92.85) | 261.92 (61.72) | 258.33 (56.47) |
| Median | 233 | 252 | 251 |
| IQR | (197;314) | (233;291) | (213;295) |
| **Hemoglobin (g/dL) (Reference: [116,** **164])** | | | |
| Mean(SD) | 127.96 (14.97) | 130.34 (11.19) | 127.97 (11.39) |
| Median | 128 | 128 | 127.5 |
| IQR | (117;140) | (124; 138.75) | (121.25; 134.5) |
| **Hematocrit (g/dL) (Reference: [0.34, 0.48])** | | | |
| Mean(SD) | 0.37.96 (0.03) | 0.37 (0.03) | 0.38 (0.02) |
| Median | 0.38 | 0.37 | 0.38 |
| IQR | (0.35;0.40) | (0.36; 0.39) | (0.36; 0.4) |
| SD: Standard Deviation. IQR: Interquartile Range. | | | |

# Appendix 2: Analysis of Supplementary Emerald Features


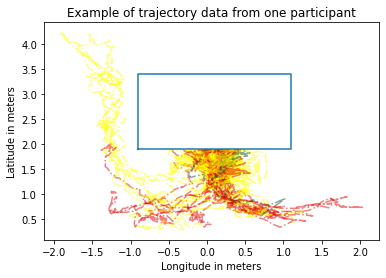
We extracted several features of speed captured in participants’ trajectories in the bedroom area including average gait speed (to assess mobility), standard deviation of gait speed (to evaluate gait variability), and total in-room traveled distance (to measure overall activity level). Note that those features were only extracted after removing the trajectory data overlapping with the bed location to target only motion due to walking (see Figure 1).

Figure 1: An example of trajectory data from one subject. Data represent location relative to the Emerald device (i.e., point (0.0) represents the location of the Emerald device). The blue rectangle represents the bed position, and the green, yellow and red lines represent the participant’s trajectories colored by gait speed range (red <0.6m/s, 0.6≤yellow<0.8, green≥0.8m/s).

Results presented in Figure 2 suggest that SLE and SjD participants exhibited slower gait speed and lower standard deviation of speed, suggesting lower physical activity than the healthy group. This finding is in-line with the results of actigraphy measures suggesting significant reduction in physical activity in disease groups when compared to healthy participants.


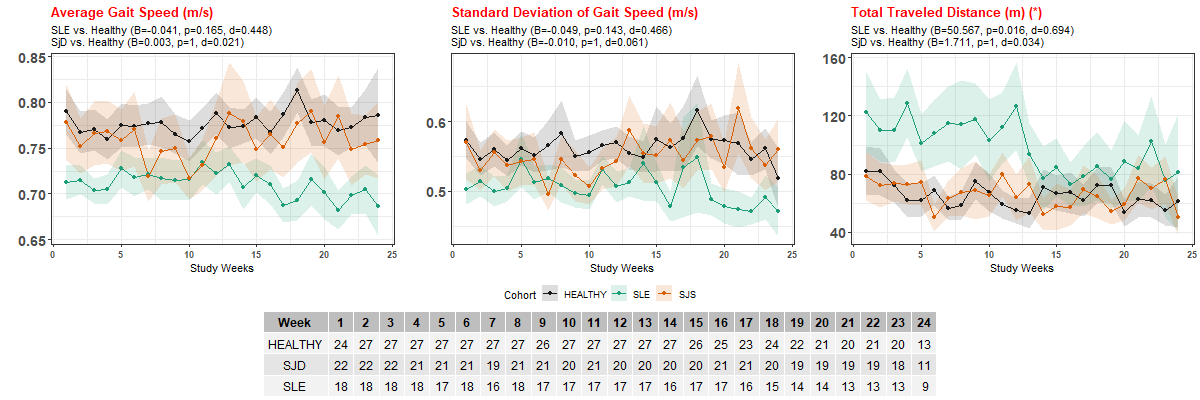


Figure 2: Additional gait features extracted from Emerald device including average gate speed, standard deviation of gait speed and the average of unique trips per day.

SLE participants were also found to have a significantly higher total in-room traveled distance in meters (B=50.56, p=0.01, d=0.69) than the healthy group. This may indicate that SLE participants spent more time in their bedrooms due to the lack of ability to perform other activities of daily living. It can also be due to more frequent awakening at night. To further investigate this, we present in Figure 3 the average distance traveled across the three groups and across the hours of the day. Results suggest that SLE participants had significantly higher in-room traveled distance, and this is consistent across all hours of the day (B=3.945, p=0.28, d=0.62). Looking at the window from midnight to 8pm, SLE participants exhibited higher activity as measured by traveled distance. This suggests more walking in the bedroom in the night due to more sleep interruptions, thus lower sleep quality. Looking at the 8am-8pm window, we see a clear distinction among SLE participants in which the traveled distance is almost constant during the day while it significantly drops for healthy and SjD subjects. This suggests that SLE participants spend more time in their bedroom, likely due the known symptoms of SLE such as fatigue, reduced physical activity, and pain that significantly interfere with the activities of daily living.


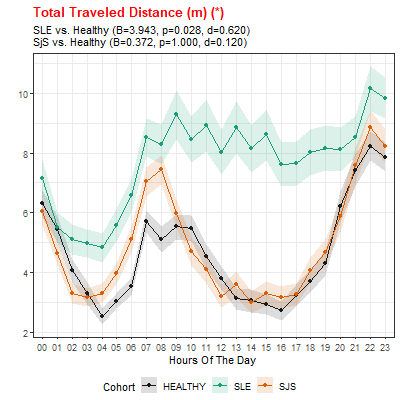


Figure 3: Average in-room total traveled distance across the three groups over the hours of the day.


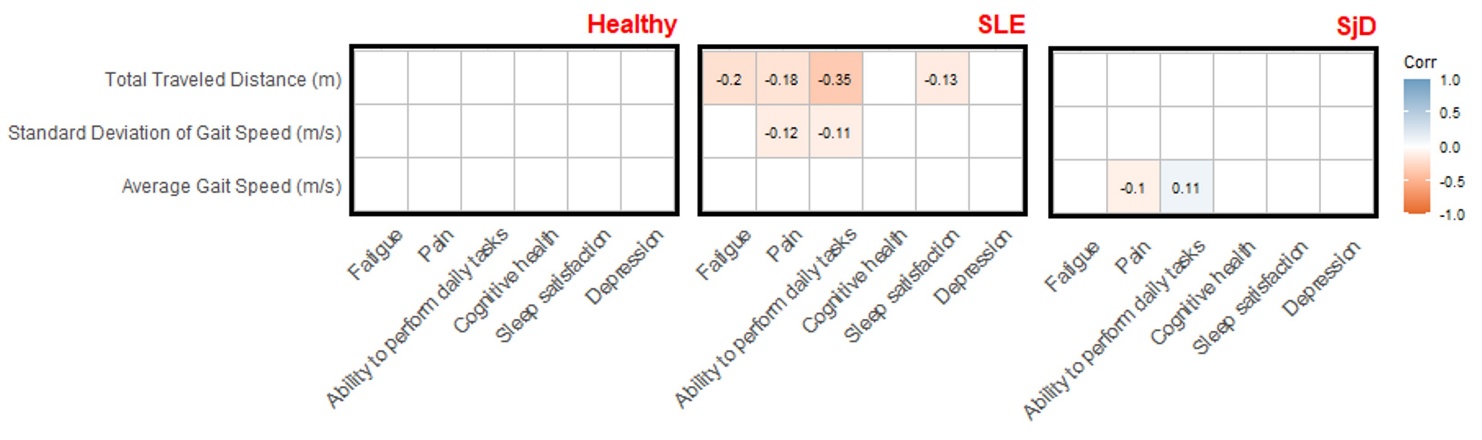


Figure 4: Repeated correlation between additional digital measures extracted from Emerald device and self-reported measures. Only significant (p<0.05) correlations are displayed.

Repeated measured correlations between the additional digital measures and the self-reported measures are displayed in Figure 4. Results are masked to display only significant correlations (i.e., p-value<0.05). It appears that for SLE patients, total traveled distance is negatively correlated with fatigue pain, ability to perform tasks and sleep satisfaction, suggesting that higher in-room traveled distance is associated with worse symptoms.

The spearman correlations between the additional digital measures and SLE and SjD disease activity suggest that none of those features are correlated with disease activity (Table 1).

Table 5: Spearman correlation between the additional digital measures with disease activity.

|  | **Correlation with SLEDAI** | | | **Correlation with ESSDAI** | | |
| --- | --- | --- | --- | --- | --- | --- |
|  | **N** | **r** | **p-value** | **N** | **r** | **p-value** |
| **Additional gait features extracted from Emerald device** | | | | | | |
| Average Gait Speed | 16 | 0.40 | 0.12 | 21 | -0.12 | 0.58 |
| Standard Deviation of Gait Speed | 16 | 0.28 | 0.28 | 21 | -0.01 | 0.95 |
| In-room Traveled Distance | 16 | 0.05 | 0.83 | 21 | 0.04 | 0.83 |

# Appendix 3: Correlation between the digital measures and individual items of disease activity scales

We examine in Appendix 3 how passively captured digital features (physical activity, sleep, and breathing metrics) relate to individual items from clinician-rated disease activity scales (i.e., SLEDAI-2K items in SLE; ESSDAI domains in SjD). Overall, item-level correlations were generally small, with a limited number of statistically significant associations. This pattern mirrors the broader literature showing that patient behavior and physiology in daily life reflect multidimensional influences—fatigue, sleep quality, pain, mood, deconditioning, medications—only partially overlapping with physician-based activity items scored at clinic visits^4–6^.

Item-specific patterns were directionally plausible. Articular and musculoskeletal involvement tended to align with lower average activity and greater fragmentation of the rest–activity cycle, consistent with prior data linking pain and functional limitations to reduced ambulatory activity and irregular diurnal patterns in SLE^7,8^. Mucocutaneous items (e.g., rash, alopecia) showed limited and inconsistent coupling to digital features, which is expected given their impact on quality of life but more indirect mechanistic links to sleep or activity^7^. Serosal involvement (e.g., pleurisy), when present, is biologically positioned to affect breathing and sleep due to chest discomfort^9^. Hematologic items (e.g., leukopenia, thrombocytopenia) showed weak cross-sectional alignment with activity or sleep, in line with evidence that laboratory cytopenias often dissociate from day-to-day symptom burden^4^. Importantly, the low prevalence of certain items (e.g., CNS, renal, or muscular manifestations, which were not observed in this cohort) inherently constrained statistical power for those domains.

The sparse cross-sectional correlations between PROs, digital phenotypes and serologies—echoes earlier work^4,5^ showing that patient-reported outcomes often track disease impact and quality of life rather than contemporaneous immunologic activity, supporting the rationale for including PROs and digital measures as complementary endpoints in trials and longitudinal care.

Table 6: Spearman correlation between the digital measures of physical activity, sleep and breathing with SLEDAI-2k individual items (part 1).

|  | Alopecia | Arthritis | Increased DNA binding | Leukopenia | Low complement | Lupus Headache | Mucosal Ulcers | Myositis |
| --- | --- | --- | --- | --- | --- | --- | --- | --- |
| **Self-reported symptoms** | | | | | | |  |  |
| Fatigue | 0.26 | 0.14 | -0.35 | -0.11 | **-0.44*** | 0.21 | -0.05 | 0.02 |
| Pain | 0.34 | 0.30 | -0.37 | -0.06 | **-0.50*** | 0.16 | -0.19 | 0.14 |
| Ability to perform daily tasks | 0.19 | 0.16 | -0.28 | 0.03 | -0.35 | 0.08 | 0.00 | 0.12 |
| Cognitive health | -0.22 | 0.04 | 0.21 | 0.25 | 0.33 | -0.14 | -0.03 | **-0.41*** |
| Sleep satisfaction | -0.37 | 0.04 | 0.28 | 0.25 | 0.41 | -0.22 | -0.06 | -0.35 |
| Depression | -0.26 | -0.11 | -0.02 | -0.08 | -0.12 | -0.06 | 0.11 | **0.41*** |
| **Physical Activity** | | | | | | | | |
| Average acceleration | 0.00 | **-0.46*** | -0.17 | -0.33 | 0.01 | 0.09 | -0.06 | 0.13 |
| Start time of the 10 most active hours | -0.03 | -0.17 | 0.08 | -0.33 | 0.26 | 0.05 | 0.20 | 0.03 |
| Start time of the 5 least active hours | -0.13 | 0.37 | 0.08 | -0.02 | -0.14 | 0.18 | -0.20 | 0.15 |
| Intra-daily variability | 0.12 | 0.26 | 0.10 | 0.33 | -0.09 | -0.09 | -0.06 | -0.10 |
| Relative amplitude | 0.03 | 0.40 | -0.22 | 0.24 | -0.24 | 0.20 | 0.04 | -0.25 |
| **Sleep** |  |  |  |  |  |  |  |  |
| Duration of being awake | -0.16 | **0.56*** | -0.04 | 0.22 | -0.23 | 0.09 | -0.538* | 0.31 |
| Duration of deep sleep | -0.37 | -0.07 | 0.37 | 0.00 | 0.53* | 0.26 | 0.02 | -0.25 |
| Duration of light sleep | -0.42 | 0.45 | 0.04 | 0.22 | -0.23 | 0.05 | 0.05 | -0.14 |
| Duration of REM sleep | -0.13 | -0.34 | 0.33 | 0.06 | 0.37 | -0.12 | 0.37 | 0.25 |
| Sleep efficiency | -0.02 | **-0.56*** | 0.08 | -0.16 | 0.23 | -0.02 | **0.60*** | -0.25 |
| Entropy of sleep stages | -0.04 | **-0.53*** | 0.16 | -0.34 | 0.30 | -0.12 | **0.60*** | -0.03 |
| Average sleep segments | -0.31 | -0.18 | 0.08 | 0.25 | 0.12 | 0.02 | 0.16 | 0.25 |
| Total duration of sleep interruptions | 0.04 | **0.67*** | -0.16 | -0.03 | -0.19 | 0.05 | -0.47 | 0.25 |
| Number of Sleep Interruptions | 0.04 | **0.58*** | 0.00 | -0.06 | -0.16 | -0.05 | -0.33 | -0.03 |
| Sleep Midpoint Time | -0.22 | 0.18 | -0.04 | -0.09 | -0.30 | 0.23 | 0.23 | 0.08 |
| Total Sleep Opportunity | -0.28 | **0.69*** | -0.04 | 0.25 | -0.19 | -0.02 | -0.33 | 0.25 |
| **Breathing** | | | | | | |  |  |
| AVG breathing rate | **0.65*** | 0.34 | -0.16 | -0.31 | -0.16 | -0.12 | 0.02 | -0.25 |
| AVG breathing when awake | **0.62*** | 0.34 | -0.12 | -0.22 | -0.26 | 0.02 | -0.02 | -0.20 |
| AVG breathing during light sleep | **0.57*** | 0.45 | -0.21 | -0.22 | -0.30 | -0.05 | 0.16 | -0.36 |
| AVG breathing during REM sleep | **0.65*** | 0.31 | -0.12 | -0.22 | -0.12 | -0.02 | -0.09 | -0.31 |
| AVG breathing during deep sleep | **0.62*** | 0.18 | -0.25 | -0.38 | -0.12 | 0.05 | 0.02 | -0.31 |
| SD breathing rate | 0.40 | **0.58*** | -0.45 | -0.34 | **-0.53*** | 0.30 | 0.02 | 0.14 |
| Approximate entropy of BBI | 0.570* | 0.40 | -0.16 | -0.09 | -0.30 | 0.02 | 0.02 | -0.20 |
| - The following items are excluded because none of those symptoms are reported by participants: Cerebrovascular Accident (CVA), Cranial Nerve Disorder, Fever, Hematuria, Organic Brain Syndrome, Pericarditis, Psychosis, Seizure, Visual Disturbance. - Correlations denoted with an Asterix (*) represent significant correlations (p-value<0.05) - Empty cells denote cases where correlation is unavailable due to zero variance in the SLEDAI item (no symptom occurrences across participants) | | | | | | | | |

Table 7: Spearman correlation between the digital measures of physical activity, sleep and breathing with SLEDAI-2k individual items (part 2).

|  | Pleurisy | Proteinuria | Pyuria | Rash | Thrombocytopenia | Urinary Casts | Vasculitis |
| --- | --- | --- | --- | --- | --- | --- | --- |
| **Self-reported symptoms** | | | | | | |  |
| Fatigue | 0.14 | 0.19 | -0.16 | -0.21 | **-0.42*** | 0.19 | 0.35 |
| Pain | 0.16 | 0.26 | -0.06 | 0.01 | -0.16 | 0.26 | 0.29 |
| Ability to perform daily tasks | 0.14 | 0.26 | 0.13 | 0.18 | -0.25 | 0.26 | -0.35 |
| Cognitive health | 0.30 | -0.23 | -0.13 | 0.01 | 0.39 | -0.23 | 0.10 |
| Sleep satisfaction | -0.15 | -0.24 | -0.32 | 0.14 | **0.50*** | -0.24 | -0.35 |
| Depression | -0.19 | -0.29 | 0.16 | 0.05 | -0.34 | -0.29 | -0.34 |
| **Physical Activity** | | | | | | | |
| Average acceleration | -0.20 | 0.16 | -0.02 | -0.08 | -0.07 | 0.16 | 0.05 |
| Start time of the 10 most active hours | -0.27 | 0.05 | 0.02 | 0.11 | 0.28 | 0.05 | 0.36 |
| Start time of the 5 least active hours | -0.10 | 0.26 | -0.36 | 0.06 | 0.20 | 0.26 | 0.02 |
| Intra-daily variability | 0.10 | -0.33 | 0.26 | -0.17 | -0.18 | -0.33 | 0.29 |
| Relative amplitude | 0.27 | 0.26 | -0.29 | 0.22 | 0.26 | 0.26 | 0.12 |
| **Sleep** |  |  |  |  |  |  |  |
| Duration of being awake | 0.00 | -0.03 | - | -0.07 | 0.05 | -0.03 | -0.36 |
| Duration of deep sleep | -0.04 | -0.14 | - | 0.07 | 0.05 | -0.14 | 0.20 |
| Duration of light sleep | -0.25 | -0.20 | - | 0.34 | -0.05 | -0.20 | -0.31 |
| Duration of REM sleep | -0.16 | -0.08 | - | 0.26 | -0.23 | -0.08 | -0.03 |
| Sleep efficiency | 0.04 | -0.03 | - | 0.01 | -0.16 | -0.03 | 0.31 |
| Entropy of sleep stages | 0.00 | 0.03 | - | 0.01 | -0.16 | 0.03 | 0.14 |
| Average sleep segments | -0.04 | -0.14 | - | -0.21 | -0.19 | -0.14 | 0.20 |
| Total duration of sleep interruptions | -0.04 | 0.08 | - | 0.12 | 0.30 | 0.08 | -0.31 |
| Number of Sleep Interruptions | -0.04 | -0.08 | - | 0.12 | 0.12 | -0.08 | -0.14 |
| Sleep Midpoint Time | 0.25 | 0.31 | - | -0.07 | -0.12 | 0.31 | -0.31 |
| Total Sleep Opportunity | 0 | -0.31 | - | 0.12 | 0.16 | -0.31 | -0.36 |
| **Breathing** | | | | | | |  |
| AVG breathing rate | 0.04 | 0.14 | - | **0.50*** | 0.43 | 0.14 | 0.25 |
| AVG breathing when awake | -0.04 | 0.25 | - | **0.56*** | 0.33 | 0.25 | 0.20 |
| AVG breathing during light sleep | 0.25 | -0.03 | - | 0.40 | 0.33 | -0.03 | 0.20 |
| AVG breathing during REM sleep | 0.16 | 0.14 | - | 0.42 | 0.43 | 0.14 | 0.25 |
| AVG breathing during deep sleep | 0.12 | 0.08 | - | 0.45 | **0.53*** | 0.08 | 0.25 |
| SD breathing rate | 0.12 | 0.20 | - | 0.29 | 0.30 | 0.20 | -0.14 |
| Approximate entropy of BBI | 0.29 | -0.03 | - | 0.29 | 0.33 | -0.03 | 0.31 |
| - The following items are excluded because none of those symptoms are reported by participants: Cerebrovascular Accident (CVA), Cranial Nerve Disorder, Fever, Hematuria, Organic Brain Syndrome, Pericarditis, Psychosis, Seizure, Visual Disturbance. - Correlations denoted with an Asterix (*) represent significant correlations (p-value<0.05) - Empty cells denote cases where correlation is unavailable due to zero variance in the SLEDAI item (no symptom occurrences across participants) | | | | | | | |

Table 8: Spearman correlation between the digital measures of physical activity, sleep and breathing with ESSDAI individual domains (part 1).

|  | Articular Score | Biological Score | Constitutional Score | Cutaneous Score | Glandular Score | Haematological Score |
| --- | --- | --- | --- | --- | --- | --- |
| **Self-reported symptoms** | | | | | | |
| Fatigue | **0.40*** | -0.33 | 0.22 | 0.02 | **0.52*** | -0.36 |
| Pain | 0.34 | -0.31 | -0.06 | -0.30 | **0.47*** | -0.35 |
| Ability to perform daily tasks | 0.21 | -0.28 | -0.09 | 0.06 | 0.07 | -0.14 |
| Cognitive health | -0.38 | 0.33 | -0.19 | 0.01 | -0.05 | 0.23 |
| Sleep satisfaction | -0.21 | 0.23 | -0.07 | -0.16 | 0.05 | **0.44*** |
| Depression | -0.10 | -0.29 | 0.03 | 0.09 | -0.31 | 0.17 |
| **Physical Activity** | | | | | | |
| Average acceleration | 0.19 | -0.23 | 0.05 | -0.10 | 0.02 | -0.20 |
| Start time of the 10 most active hours | -0.06 | 0.17 | -0.06 | 0.12 | -0.05 | -0.19 |
| Start time of the 5 least active hours | -0.23 | 0.04 | -0.16 | 0.09 | **-0.38*** | 0.09 |
| Intra-daily variability | 0.06 | 0.25 | 0.09 | -0.29 | 0.26 | 0.07 |
| Relative amplitude | 0.08 | 0.12 | -0.29 | 0.15 | 0.02 | -0.17 |
| **Sleep** |  |  |  |  |  |  |
| Duration of being awake | -0.13 | 0.15 | 0.04 | -0.07 | 0.00 | -0.03 |
| Duration of deep sleep | 0.06 | 0.19 | 0.00 | -0.09 | -0.15 | -0.19 |
| Duration of light sleep | 0.25 | 0.07 | -0.26 | -0.17 | 0.07 | 0.00 |
| Duration of REM sleep | 0.26 | 0.26 | -0.16 | -0.13 | -0.13 | -0.03 |
| Sleep efficiency | 0.28 | -0.07 | -0.10 | 0.04 | 0.04 | 0.05 |
| Entropy of sleep stages | 0.03 | 0.15 | -0.20 | -0.30 | -0.17 | -0.19 |
| Average sleep segments | 0.11 | -0.30 | -0.12 | -0.20 | 0.11 | -0.03 |
| Total duration of sleep interruptions | 0.01 | 0.19 | 0.34 | 0.28 | 0.06 | -0.19 |
| Number of Sleep Interruptions | 0.03 | 0.26 | 0.08 | 0.03 | -0.15 | 0.00 |
| Sleep Midpoint Time | -0.28 | -0.04 | -0.18 | 0.02 | 0.15 | -0.30 |
| Total Sleep Opportunity | 0.22 | 0.07 | -0.06 | -0.13 | 0.06 | -0.03 |
| **Breathing** | | | | | | |
| AVG breathing rate | 0.28 | 0.07 | **0.48*** | -0.24 | 0.30 | -0.08 |
| AVG breathing when awake | 0.30 | 0.04 | **0.50*** | -0.15 | 0.26 | -0.11 |
| AVG breathing during light sleep | 0.28 | 0.26 | 0.34 | -0.26 | 0.17 | -0.11 |
| AVG breathing during REM sleep | 0.28 | 0.04 | 0.42 | -0.22 | 0.33 | -0.13 |
| AVG breathing during deep sleep | 0.26 | 0.00 | 0.38 | -0.28 | **0.44*** | -0.19 |
| SD breathing rate | 0.16 | 0.22 | 0.22 | -0.02 | 0.39 | -0.16 |
| Approximate entropy of BBI | 0.24 | 0.04 | 0.28 | -0.20 | 0.37 | -0.21 |
| - The following items are excluded because none of those symptoms are reported by participants: CNS Score, Renal Score, Muscular Score. - Correlations denoted with an Asterix (*) represent significant correlations (p-value<0.05) | | | | | | |

Table 9: Spearman correlation between the digital measures of physical activity, sleep and breathing with ESSDAI individual domains (part 2).

|  | Articular Score | Glandular Score | Haematological Score |
| --- | --- | --- | --- |
| **Self-reported symptoms** | | | |
| Fatigue | 0.01 | 0.26 | -0.17 |
| Pain | 0.10 | 0.21 | -0.12 |
| Ability to perform daily tasks | -0.10 | -0.20 | -0.28 |
| Cognitive health | -0.01 | -0.10 | 0.12 |
| Sleep satisfaction | -0.20 | -0.01 | 0.15 |
| Depression | -0.03 | -0.39 | -0.07 |
| **Physical Activity** | | | |
| Average acceleration | -0.07 | -0.02 | -0.01 |
| Start time of the 10 most active hours | 0.12 | 0.20 | 0.09 |
| Start time of the 5 least active hours | 0.29 | 0.06 | -0.12 |
| Intra-daily variability | -0.15 | 0.18 | 0.20 |
| Relative amplitude | 0.15 | -0.08 | -0.25 |
| **Sleep** |  |  |  |
| Duration of being awake | 0.04 | -0.06 | -0.22 |
| Duration of deep sleep | -0.30 | -0.06 | -0.22 |
| Duration of light sleep | -0.24 | **0.47*** | 0.26 |
| Duration of REM sleep | -0.28 | 0.21 | 0.07 |
| Sleep efficiency | -0.20 | 0.22 | 0.26 |
| Entropy of sleep stages | -0.24 | 0.02 | -0.22 |
| Average sleep segments | 0.07 | 0.20 | 0.04 |
| Total duration of sleep interruptions | 0.19 | -0.06 | -0.30 |
| Number of Sleep Interruptions | -0.27 | -0.12 | -0.11 |
| Sleep Midpoint Time | 0.39 | -0.01 | -0.26 |
| Total Sleep Opportunity | -0.28 | 0.34 | 0.11 |
| **Breathing** | | | |
| AVG breathing rate | -0.19 | -0.17 | -0.37 |
| AVG breathing when awake | -0.09 | -0.10 | -0.37 |
| AVG breathing during light sleep | -0.20 | -0.19 | -0.37 |
| AVG breathing during REM sleep | -0.17 | -0.16 | -0.37 |
| AVG breathing during deep sleep | -0.11 | -0.08 | -0.37 |
| SD breathing rate | 0.07 | 0.01 | -0.30 |
| Approximate entropy of BBI | -0.09 | -0.11 | -0.37 |
| - The following items are excluded because none of those symptoms are reported by participants: CNS Score, Renal Score, Muscular Score. - Correlations denoted with an Asterix (*) represent significant correlations (p-value<0.05) | | | |

# Appendix 4: Correlation between the digital measures and Lab measurements at baseline

Results presented in Appendix 4 show that most cross-sectional correlations between passively captured digital measures (activity, sleep, breathing) and laboratory indices at baseline are small, suggesting these modalities provide complementary—not redundant—information about disease impact in systemic autoimmunity^4,10^. A focused set of associations appears biologically plausible: fatigue positively correlated with hemoglobin and hematocrit; later timing of peak daily activity was associated with lower lymphocyte counts; longer light-sleep duration related to higher C3; lower sleep satisfaction related to higher platelets; and higher average respiratory rate (overall, awake, and during REM) correlated with higher lymphocyte counts, while reduced beat-to-breath interval complexity related to lower red blood cell counts. Together, these patterns are consistent with literature linking sleep–wake regulation^9,11^ and autonomic–respiratory control^12^ to immune function, as well as with the known multidimensional determinants of fatigue and activity in rheumatic disease^7,8,13^.

Table 10: Correlation between the digital measures (average first 4 weeks) and Lab measurements at baseline for SLE participants.

|  | Anti‑dsDNA | C3 | C4 | Hct | Hb | IgG | LYM | PLT | RBC |
| --- | --- | --- | --- | --- | --- | --- | --- | --- | --- |
| **Self-reported symptoms** | | | | | | | | | |
| Fatigue | -0.03 | **0.50*** | **0.46*** | 0.37 | **0.38*** | **-0.43*** | **0.40*** | **0.50*** | -0.24 |
| Pain | -0.18 | **0.55*** | **0.52*** | 0.28 | 0.24 | **-0.46*** | **0.42*** | **0.50*** | -0.19 |
| Ability to perform daily tasks | 0.03 | 0.35 | 0.17 | 0.25 | 0.22 | -0.02 | 0.10 | 0.28 | 0.00 |
| Cognitive health | 0.18 | **-0.43*** | **-0.43*** | -0.30 | **-0.37*** | 0.11 | -0.12 | -0.14 | 0.00 |
| Sleep satisfaction | -0.07 | -0.14 | -0.22 | -0.06 | -0.11 | 0.14 | -0.20 | -0.13 | **0.44*** |
| Depression | -0.01 | 0.36 | 0.24 | 0.21 | 0.29 | 0.11 | -0.04 | 0.16 | 0.15 |
| **Physical Activity** | | | | | | | | |  |
| Average acceleration | -0.25 | -0.22 | -0.12 | 0.18 | 0.19 | -0.12 | 0.06 | 0.09 | 0.27 |
| Start time of the 10 most active hours | -0.27 | 0.22 | 0.21 | 0.11 | 0.05 | -0.15 | 0.00 | 0.24 | 0.43* |
| Start time of the 5 least active hours | -0.25 | 0.31 | 0.17 | -0.02 | -0.02 | -0.26 | 0.27 | -0.04 | 0.26 |
| Intra-daily variability | 0.16 | 0.16 | 0.10 | 0.11 | 0.13 | 0.13 | -0.04 | 0.05 | **-0.55*** |
| Relative amplitude | 0.00 | 0.01 | -0.08 | -0.14 | -0.18 | -0.10 | -0.04 | -0.09 | -0.06 |
| **Sleep** | | | | | | | | | |
| Duration of being awake | -0.05 | 0.42 | 0.06 | -0.03 | -0.16 | 0.21 | 0.00 | -0.17 | -0.02 |
| Duration of deep sleep | **0.48*** | -0.32 | -0.25 | -0.17 | -0.01 | 0.30 | -0.40 | 0.10 | 0.00 |
| Duration of light sleep | 0.35 | -0.02 | -0.32 | -0.24 | -0.25 | 0.20 | -0.46 | 0.12 | -0.17 |
| Duration of REM sleep | 0.30 | -0.37 | -0.11 | -0.32 | -0.18 | 0.10 | **-0.57*** | 0.02 | -0.07 |
| Sleep efficiency | 0.19 | -0.46 | -0.14 | -0.03 | 0.13 | -0.17 | -0.13 | 0.23 | -0.04 |
| Entropy of sleep stages | -0.03 | -0.39 | -0.08 | 0.08 | 0.24 | -0.01 | -0.04 | 0.02 | 0.10 |
| Average sleep segments | 0.32 | -0.20 | 0.15 | -0.25 | -0.15 | 0.07 | **-0.63*** | -0.08 | 0.00 |
| Total duration of sleep interruptions | 0.21 | 0.16 | -0.07 | -0.08 | -0.23 | 0.32 | -0.17 | -0.14 | -0.07 |
| Number of Sleep Interruptions | -0.07 | 0.30 | -0.18 | 0.04 | -0.05 | 0.07 | 0.25 | 0.09 | -0.08 |
| Sleep Midpoint Time | 0.20 | 0.03 | -0.26 | 0.11 | 0.14 | 0.03 | -0.09 | 0.18 | -0.21 |
| Total Sleep Opportunity | 0.15 | 0.19 | -0.07 | -0.14 | -0.31 | 0.27 | -0.27 | -0.16 | -0.11 |
| **Breathing** | | | | | | |  |  |  |
| AVG breathing rate | -0.34 | 0.22 | -0.06 | 0.26 | 0.21 | -0.40 | **0.54*** | -0.04 | -0.30 |
| AVG breathing when awake | -0.32 | 0.28 | -0.08 | 0.26 | 0.25 | -0.38 | **0.49*** | -0.07 | -0.34 |
| AVG breathing during light sleep | -0.26 | 0.10 | -0.07 | 0.29 | 0.19 | -0.34 | 0.41 | -0.09 | -0.28 |
| AVG breathing during REM sleep | -0.30 | 0.18 | -0.10 | 0.17 | 0.12 | -0.37 | **0.50*** | -0.03 | -0.29 |
| AVG breathing during deep sleep | -0.26 | 0.07 | -0.11 | 0.16 | 0.09 | -0.24 | 0.41 | -0.09 | -0.25 |
| SD breathing rate | -0.09 | 0.10 | -0.09 | 0.22 | 0.08 | -0.25 | 0.21 | -0.21 | -0.33 |
| Approximate entropy of BBI | -0.23 | 0.07 | -0.24 | 0.19 | 0.13 | -0.35 | 0.36 | -0.14 | **-0.50*** |
| - Correlations denoted with an Asterix (*) represent significant correlations (p-value<0.05) - Anti‑dsDNA:Anti–double‑stranded DNA, C3: Complement C3, C4: Complement C4, Hct: Hematocrit, Hb: Hemoglobin, IgG: Immunoglobulin G, LYM: Lymphocytes, PLT: Platelets, RBC: Red Blood Cells (Erythrocytes). | | | | | | | | | |

Table 11: Correlation between the digital measures (average first 4 weeks) and Laboratory measurements at baseline for SjD participants.

|  | Anti‑dsDNA | C3 | C4 | Hct | Hb | IgG | LYM | PLT | RBC |
| --- | --- | --- | --- | --- | --- | --- | --- | --- | --- |
| **Self-reported symptoms** | | | | | | |  |  | |
| Fatigue | - | - | - | **0.44*** | **0.52*** | - | 0.17 | 0.17 | 0.35 |
| Pain | - | - | - | 0.35 | **0.44*** | - | 0.30 | 0.11 | 0.21 |
| Ability to perform daily tasks | - | - | - | 0.23 | 0.31 | - | -0.16 | 0.09 | 0.33 |
| Cognitive health | - | - | - | -0.17 | -0.25 | - | -0.27 | -0.34 | 0.10 |
| Sleep satisfaction | - | - | - | -0.21 | -0.22 | - | -0.05 | **-0.49*** | -0.18 |
| Depression | - | - | - | -0.01 | 0.01 | - | -0.06 | 0.27 | -0.18 |
| **Physical Activity** | | | | | | | | | |
| Average acceleration | - | - | - | 0.01 | 0.03 | - | -0.14 | 0.02 | 0.07 |
| Start time of the 10 most active hours | - | - | - | 0.05 | -0.02 | - | **-0.40*** | -0.19 | 0.17 |
| Start time of the 5 least active hours | - | - | - | -0.18 | -0.15 | - | -0.15 | -0.16 | -0.22 |
| Intra-daily variability | - | - | - | 0.04 | 0.02 | - | -0.15 | -0.07 | -0.05 |
| Relative amplitude | - | - | - | 0.18 | 0.19 | - | 0.34 | 0.07 | -0.05 |
| **Sleep** | | | | | | | | | |
| Duration of being awake | - | - | - | **0.56*** | 0.41 | - | -0.12 | 0.25 | 0.14 |
| Duration of deep sleep | - | - | - | -0.33 | -0.22 | - | -0.01 | **-0.47*** | -0.02 |
| Duration of light sleep | - | - | - | -0.02 | 0.08 | - | 0.06 | 0.01 | 0.22 |
| Duration of REM sleep | - | - | - | -0.23 | -0.23 | - | 0.23 | -0.36 | -0.24 |
| Sleep efficiency | - | - | - | -0.42 | -0.28 | - | 0.14 | -0.28 | -0.08 |
| Entropy of sleep stages | - | - | - | 0.04 | -0.01 | - | 0.01 | -0.41 | -0.13 |
| Average sleep segments | - | - | - | -0.26 | -0.15 | - | 0.17 | -0.01 | 0.01 |
| Total duration of sleep interruptions | - | - | - | 0.33 | 0.23 | - | -0.25 | 0.27 | 0.10 |
| Number of Sleep Interruptions | - | - | - | 0.34 | 0.22 | - | -0.19 | -0.10 | -0.04 |
| Sleep Midpoint Time | - | - | - | 0.28 | 0.27 | - | 0.00 | 0.31 | **0.45*** |
| Total Sleep Opportunity | - | - | - | 0.25 | 0.25 | - | 0.02 | -0.01 | 0.03 |
| **Breathing** | | | | | | | | | |
| AVG breathing rate | - | - | - | -0.02 | -0.06 | - | 0.26 | -0.03 | -0.05 |
| AVG breathing when awake | - | - | - | 0.06 | 0.08 | - | 0.27 | 0.07 | 0.07 |
| AVG breathing during light sleep | - | - | - | -0.06 | -0.14 | - | 0.22 | -0.08 | -0.16 |
| AVG breathing during REM sleep | - | - | - | -0.06 | -0.10 | - | 0.27 | -0.01 | -0.10 |
| AVG breathing during deep sleep | - | - | - | -0.06 | -0.05 | - | 0.33 | -0.02 | 0.02 |
| SD breathing rate | - | - | - | 0.45 | 0.36 | - | 0.41 | 0.20 | 0.02 |
| Approximate entropy of BBI | - | - | - | 0.24 | 0.21 | - | 0.29 | 0.17 | 0.10 |
| - Correlations denoted with an Asterix (*) represent significant correlations (p-value<0.05) - Anti‑dsDNA:Anti–double‑stranded DNA, C3: Complement C3, C4: Complement C4, Hct: Hematocrit, Hb: Hemoglobin, IgG: Immunoglobulin G, LYM: Lymphocytes, PLT: Platelets, RBC: Red Blood Cells (Erythrocytes). - Empty cells denote cases where correlation is unavailable due laboratory test not administered for this cohort. | | | | | | | | | |

Table 12: Correlation between the digital measures (average first 4 weeks) and Laboratory measurements at baseline for HNV participants.

|  | Anti‑dsDNA | C3 | C4 | Hct | Hb | IgG | LYM | PLT | RBC |
| --- | --- | --- | --- | --- | --- | --- | --- | --- | --- |
| **Self-reported symptoms** | | | | | | |  |  | |
| Fatigue | - | - | - | -0.11 | -0.01 | - | -0.03 | 0.06 | 0.01 |
| Pain | - | - | - | -0.07 | 0.01 | - | -0.01 | 0.01 | 0.03 |
| Ability to perform daily tasks | - | - | - | 0.00 | 0.05 | - | -0.23 | 0.23 | 0.12 |
| Cognitive health | - | - | - | 0.22 | 0.17 | - | 0.04 | -0.32 | -0.07 |
| Sleep satisfaction | - | - | - | 0.23 | 0.12 | - | 0.12 | -0.12 | 0.09 |
| Depression | - | - | - | 0.02 | 0.04 | - | -0.08 | 0.13 | -0.06 |
| **Physical Activity** | | | | | | | | | |
| Average acceleration | - | - | - | 0.07 | 0.08 | - | -0.09 | -0.04 | 0.03 |
| Start time of the 10 most active hours | - | - | - | -0.10 | 0.06 | - | 0.18 | -0.15 | -0.01 |
| Start time of the 5 least active hours | - | - | - | -0.17 | -0.17 | - | 0.24 | -0.07 | 0.28 |
| Intra-daily variability | - | - | - | 0.23 | 0.27 | - | 0.05 | -0.28 | 0.02 |
| Relative amplitude | - | - | - | -0.15 | -0.21 | - | 0.00 | 0.13 | 0.07 |
| **Sleep** | | | | | | | | | |
| Duration of being awake | - | - | - | 0.01 | 0.07 | - | -0.26 | -0.16 | -0.01 |
| Duration of deep sleep | - | - | - | -0.13 | 0.03 | - | 0.37 | -0.05 | -0.04 |
| Duration of light sleep | - | - | - | -0.25 | -0.17 | - | 0.12 | -0.10 | -0.14 |
| Duration of REM sleep | - | - | - | 0.11 | 0.15 | - | 0.36 | -0.07 | 0.05 |
| Sleep efficiency | - | - | - | 0.02 | -0.02 | - | 0.34 | 0.10 | 0.00 |
| Entropy of sleep stages | - | - | - | 0.03 | 0.16 | - | 0.03 | 0.16 | 0.06 |
| Average sleep segments | - | - | - | 0.07 | -0.03 | - | 0.17 | 0.22 | **-0.41*** |
| Total duration of sleep interruptions | - | - | - | -0.23 | -0.18 | - | 0.03 | -0.12 | 0.13 |
| Number of Sleep Interruptions | - | - | - | -0.14 | 0.06 | - | 0.00 | -0.24 | 0.17 |
| Sleep Midpoint Time | - | - | - | 0.00 | 0.06 | - | 0.28 | -0.11 | 0.07 |
| Total Sleep Opportunity | - | - | - | -0.27 | -0.07 | - | 0.16 | 0.01 | -0.11 |
| **Breathing** | | | | | | | | | |
| AVG breathing rate | - | - | - | -0.36 | -0.21 | - | 0.03 | 0.13 | 0.06 |
| AVG breathing when awake | - | - | - | -0.25 | -0.10 | - | 0.10 | 0.04 | 0.25 |
| AVG breathing during light sleep | - | - | - | -0.30 | -0.19 | - | -0.09 | 0.06 | 0.04 |
| AVG breathing during REM sleep | - | - | - | -0.36 | -0.22 | - | 0.02 | 0.14 | 0.04 |
| AVG breathing during deep sleep | - | - | - | -0.39 | -0.26 | - | 0.05 | 0.15 | 0.04 |
| SD breathing rate | - | - | - | -0.09 | -0.09 | - | -0.21 | -0.27 | 0.21 |
| Approximate entropy of BBI | - | - | - | -0.35 | -0.26 | - | -0.06 | 0.15 | 0.11 |
| - Correlations denoted with an Asterix (*) represent significant correlations (p-value<0.05) - Anti‑dsDNA:Anti–double‑stranded DNA, C3: Complement C3, C4: Complement C4, Hct: Hematocrit, Hb: Hemoglobin, IgG: Immunoglobulin G, LYM: Lymphocytes, PLT: Platelets, RBC: Red Blood Cells (Erythrocytes). - Empty cells denote cases where correlation is unavailable due laboratory test not administered for this cohort. | | | | | | | | | |

# References

1. Schimmer BP, Funder JW. ACTH, adrenal steroids, and pharmacology of the adrenal cortex. *Goodman Gilman’s Pharmacol Basis Ther*. Published online 2011:1209-1235.

2. Liu D, Ahmet A, Ward L, et al. A practical guide to the monitoring and management of the complications of systemic corticosteroid therapy. *Allergy Asthma Clin Immunol*. 2013;9(1):30.

3. Bancos I, Hahner S, Tomlinson J, Arlt W. Diagnosis and management of adrenal insufficiency. *Lancet Diabetes Endocrinol*. 2015;3(3):216-226.

4. Parra Sánchez AR, Bultink IEM, Twisk JWR, van Vollenhoven RF, Voskuyl AE, Tsang-A-Sjoe MWP. Patient–physician discordance in assessing SLE disease activity: longitudinal analysis of disease-related variables and quality of life. *Rheumatology*. 2026;65(2):keaf653. doi:10.1093/rheumatology/keaf653

5. Fanouriakis A, Kostopoulou M, Andersen J, et al. EULAR recommendations for the management of systemic lupus erythematosus: 2023 update. *Ann Rheum Dis*. 2024;83(1):15-29. doi:10.1136/ard-2023-224762

6. Ramos-Casals M, Brito-Zerón P, Bombardieri S, et al. EULAR recommendations for the management of Sjögren’s syndrome with topical and systemic therapies. *Ann Rheum Dis*. 2020;79(1):3-18. doi:10.1136/annrheumdis-2019-216114

7. Iaboni A, Ibanez D, Gladman DD, Urowitz MB, Moldofsky H. Fatigue in systemic lupus erythematosus: contributions of disordered sleep, sleepiness, and depression. *J Rheumatol*. 2006;33(12):2453-2457.

8. Kawka L, Schlencker A, Mertz P, Martin T, Arnaud L. Fatigue in Systemic Lupus Erythematosus: An Update on Its Impact, Determinants and Therapeutic Management. *J Clin Med*. 2021;10(17):3996. doi:10.3390/jcm10173996

9. Besedovsky L, Lange T, Born J. Sleep and immune function. *Pflugers Arch*. 2012;463(1):121-137. doi:10.1007/s00424-011-1044-0

10. Izmailova ES, Wagner JA, Perakslis ED. Wearable Devices in Clinical Trials: Hype and Hypothesis. *Clin Pharmacol Ther*. 2018;104(1):42-52. doi:10.1002/cpt.966

11. Scheiermann C, Kunisaki Y, Frenette PS. Circadian control of the immune system. *Nat Rev Immunol*. 2013;13(3):190-198. doi:10.1038/nri3386

12. Davies K, Ng WF. Autonomic Nervous System Dysfunction in Primary Sjögren’s Syndrome. *Front Immunol*. 2021;12:702505. doi:10.3389/fimmu.2021.702505

13. Dardin LP, Garcia ABA, Gazoni FM, Santos FCD, Mello MT de, Trevisani VFM. Correlation of sleep quality with fatigue and disease activity among patients with primary Sjögren’s syndrome: a cross-sectional study. *Sao Paulo Med J Rev Paul Med*. 2020;138(2):146-151. doi:10.1590/1516-3180.2019.0251.R1.1912019
